# Imaging the distribution of skin lipids and topically applied compounds in human skin using mass spectrometry

**DOI:** 10.1038/s41598-018-34286-x

**Published:** 2018-11-12

**Authors:** Peter Sjövall, Lisa Skedung, Sébastien Gregoire, Olga Biganska, Franck Clément, Gustavo S. Luengo

**Affiliations:** 1 RISE Research Institutes of Sweden, Chemistry and Materials, SE-50115 Borås, Sweden; 2 RISE Research Institutes of Sweden, Surface, Process and Formulation, SE-11428 Stockholm, Sweden; 3 L’OREAL Research and Innovation, 93601 Aulnay-sous-Bois, France; 4 L’OREAL Research and Innovation, 94550 Chevilly-Larue, France

## Abstract

The barrier functions of skin against water loss, microbial invasion and penetration of xenobiotics rely, in part, on the spatial distribution of the biomolecular constituents in the skin structure, particularly its horny layer (stratum corneum). However, all skin layers are important to describe normal and dysfunctional skin conditions, and to develop adapted therapies or skin care products. In this work, time-of-flight secondary ion mass spectrometry (ToF-SIMS) combined with scanning electron microscopy (SEM) was used to image the spatial distribution of a variety of molecular species, from stratum corneum down to dermis, in cross-section samples of human abdominal skin. The results demonstrate the expected localization of ceramide and saturated long-chain fatty acids in stratum corneum (SC) and cholesterol sulfate in the upper part of the viable epidermis. The localization of exogenous compounds is demonstrated by the detection and imaging of carvacrol (a constituent of oregano or thyme essential oil) and ceramide, after topical application onto *ex vivo* human skin. Carvacrol showed pronounced accumulation to triglyceride-containing structures in the deeper parts of dermis. In contrast, the exogenous ceramide was found to be localized in SC. Furthermore, the complementary character of this approach with classical *ex vivo* skin absorption analysis methods is demonstrated.

## Introduction

The mammalian skin is a highly complex organ with a variety of advanced functions, one of the most important being to act as a barrier to excessive water loss, and xenobiotic and microbial assaults. A properly functioning barrier is essential for a healthy skin, but it also constitutes a challenge for the development of pharmaceutical and active cosmetic formulations, in which penetration of active compounds is crucial. Yet, for many cosmetic products such as UV filters, hair dyes, and hygiene products, penetration of the skin barrier is not desired and should be prevented. Other important functions of the skin include heat and tactile sensing, thermoregulation (sweat glands), UV protection (pigment cells) and immune response. Furthermore, the skin provides mechanical protection of the body, requiring both a hard surface and a soft, elastic connective tissue underneath^1^.

The human skin varies in thickness according to body locations and is comprised of three distinct basic layers: the hypodermis, the dermis and the epidermis. Whereas the fat-rich hypodermis attaches to the underlying muscular tissue and provides padding and insulation for the body, dermis is composed of a dense connective tissue dominated by networks of collagen and elastin fibers within an extrafibrillar matrix of glucosaminoglycans and proteoglycans, giving strength and elasticity to the skin. Dermis also contains sensory organs, nerve endings, blood vessels, sweat glands, sebaceous glands, and hair follicle roots^1^. In contrast to dermis, epidermis lacks vascular blood supply and is dominated by keratinocytes, which continuously migrate from the lower epidermal layer (stratum basale) under multiple differentiation stages towards the topmost epidermal layer, the stratum corneum (SC), where they are terminally transformed into flattened, keratin-rich, non-viable (dead) cells called corneocytes. The corneocytes in SC are interconnected by proteins called corneodesmosomes and arranged in single cell layers (10–25 layers in human skin), of which the outer ones are constantly removed from the skin surface in a process called desquamation, in order to complete the continuously ongoing skin regeneration process. Furthermore, the corneocytes are coated with a cross-linked protein layer, the cornified envelope, onto which a lipid layer is covalently bound^2,3^.

The barrier properties of the skin are maintained by the unique molecular architecture of SC, in which the corneocyte layers are embedded in a structurally well-organized matrix of intercellular lipids, sometimes referred to as the “bricks-in-mortar” structure^2–6^. The composition of the lipid matrix is unique to SC and contains approximately 50% ceramides, 25% cholesterol and 10–20% free fatty acids^4,6,7^, where the latter are dominated by saturated long-chain fatty acids, mainly C24:0 and C26:0^8,9^, and the ceramides are composed of at least 13 different subtypes, each with its own distribution of fatty acyl chain lengths, of which the major ones are also those containing 24 or 26 carbon atoms^6,10^. The lipids are organized in stacked bilayer structures, showing well-defined periodicities of 13 nm (long periodicity phase) and 6 nm (short periodicity phase)^2,7,11^. Importantly, it has been shown that disturbances in the lipid composition and/or organization can be related to changes in the barrier properties and to a range of skin diseases, including atopic dermatitis, lamellar ichthyosis and psoriasis^3,6,9,12^. The supply of lipids to SC is generated by so called lamellar bodies (LB), which are vesicular particles found at high concentrations in keratinocytes of the upper viable epidermis (stratum granulosum, SG). The LBs contain precursors of the SC lipids, which are released by exocytosis into the intercellular space at the SG/SC interface, together with enzymes for their final processing^5,6^. Another important skin lipid is cholesterol sulfate, which is believed to be involved in the keratinocyte differentiation and desquamation process. Cholesterol sulfate is primarily located to the upper part of the viable epidermis, with only low concentrations in SC^13,14^.

Passive penetration through SC has been found to depend critically on molecular weight, with the penetration being drastically reduced for molecules larger than 400–500 Da, and on the lipophilic properties of the substances^15^. Different strategies have therefore been investigated to assist the delivery of pharmaceutical and cosmetic ingredients into the skin, including advanced formulations^16^, penetration enhancers^17^, chemical and physical methods to overcome the barrier (such as microneedles, ultrasound or electric cavitation)^18^. A common method to study the molecular penetration is to monitor the diffusion through the skin in a so-called Franz cell, in combination with analysis of different separated skin layers using, for example, mass spectrometry methods. This approach provides highly sensitive and quantitative concentration data, but the spatial information is limited to the various skin layers that can be isolated. Furthermore, various imaging techniques have been applied to obtain better spatial information, including CLSM^19^, FTIR^20,21^, Raman^22,23^, CARS^24^. Although these methods provide high spatial resolution, their sensitivities and chemical specificities are limited, which leads to uncertainties regarding the interpretation of the recorded images.

Imaging mass spectrometry methods provide high chemical specificity and have also been used for skin analysis. Using MALDI imaging, a selection of endogenous lipids have been identified and mapped in skin cross sections^25–30^ and, recently, the penetration of four drug molecules was successfully monitored^31^. Time-of-flight secondary ion mass spectrometry (ToF-SIMS) is another imaging mass spectrometry technique that has been used successfully for biomolecular imaging of cells and tissues^32–35^. The main advantage of ToF-SIMS is the spatial resolution, which is routinely in the submicrometer range, as compared to typically 10–50 µm for MALDI imaging, and the fact that no matrix deposition is needed for ToF-SIMS analysis. However, relatively few studies have been reported for ToF-SIMS analysis of skin^36–43^. Kezutyte *et al*.^39^ and, more recently, Cizinauskas *et al*.^36,37^ used ToF-SIMS to study the effect of fatty acids and natural oils as penetration enhancers, while Starr *et al*.^43^ was able to characterize age-related changes in the abundance and spatial distribution of SC lipids by ToF-SIMS analysis of tape stripped skin samples. Whereas the spatial resolution is better for ToF-SIMS, the sensitivity is generally higher for MALDI imaging, as recently concluded from a MALDI imaging study of drug penetration^31^, which could be directly compared to a previous ToF-SIMS study^42^.

In this paper, ToF-SIMS was used to obtain unprecedented molecular imaging data of the major skin lipids together with the distribution of two topically applied exogenous compounds, carvacrol and ceramide. Whereas carvacrol is the main component of oregano and thyme essential oil (and has also been reported to be an efficient penetration enhancer^17^), ceramide is used in various topical formulations with the purpose to restore or maintain the lipid structure and composition in SC^21^, thereby alleviating problems with defective barrier function and dry skin. The ToF-SIMS analysis was combined with cutaneous absorption analysis using GC-MS and LC-MS, to obtain quantitative penetration data, and SEM imaging for structural correlation. The results demonstrate the simultaneous imaging of exogenous compounds and the endogenous lipid components in human skin cross sections at micrometre spatial resolution. This approach allows not only for analysis of molecular penetration, but also for monitoring the skin status in terms of lipid composition and distributions, as well as the effect of exogenous compounds on the endogenous lipid composition and distributions.

## Results

### Mapping of endogenous lipids in skin cross sections

**Figure 1:**
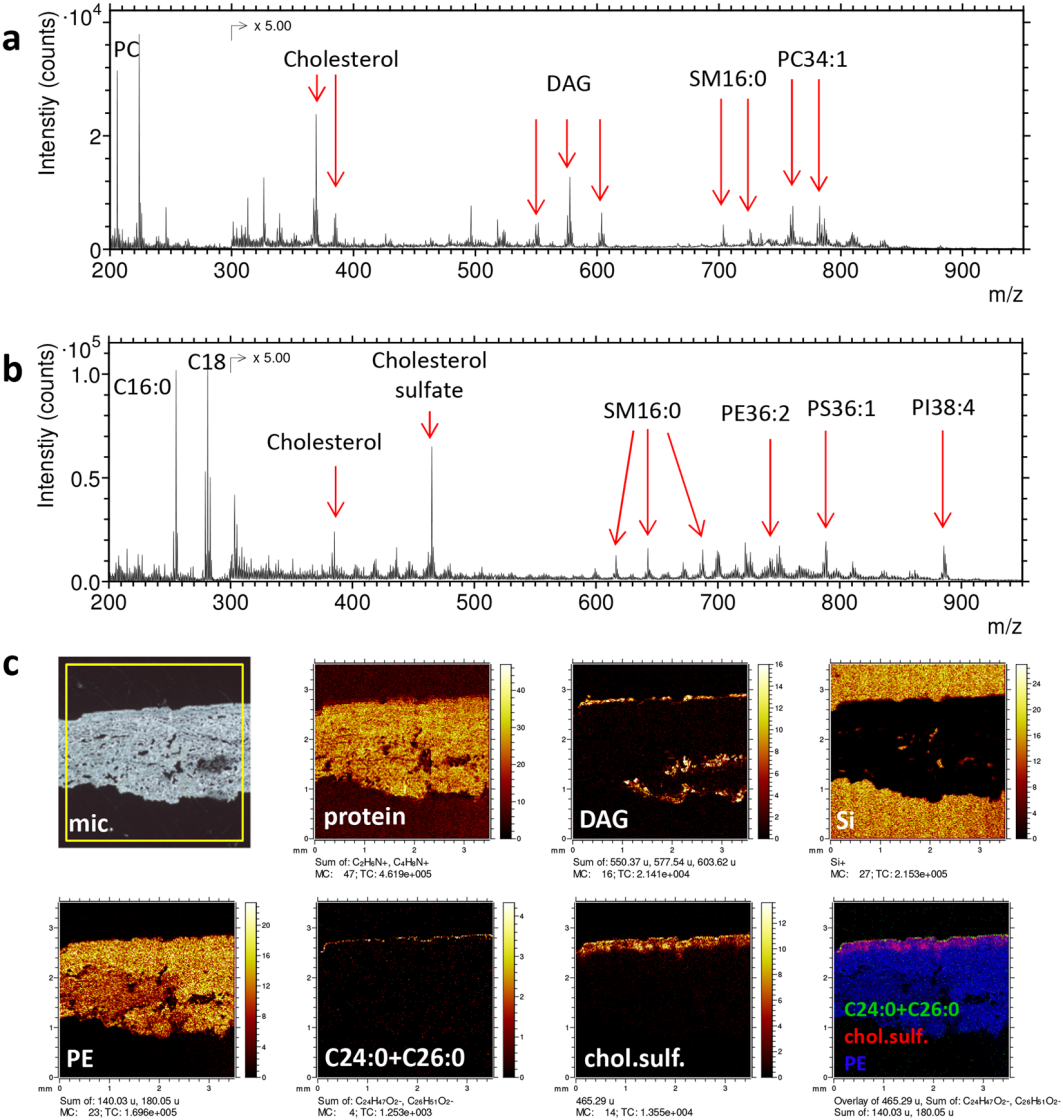
Large-area ToF-SIMS data of skin cross section. (**a**) Positive and (**b**) negative ion spectra. (**c**) Optical micrograph (mic), with the analysis area (3.5 × 3.5 mm^2^) indicated by the yellow square, positive ion images of protein fragments (m/z 44 + 70), DAG (m/z (549–551) + (575–579) + (601–605)), representing triglycerides, silicon (m/z 28), and negative ion images of PE fragments (m/z 140 + 180), C24:0 + C26:0 fatty acids (m/z 367 + 395), cholesterol sulfate (m/z 465), and a 3-colour overlay image of C24:0 + C26:0 (green), cholesterol sulfate (red) and PE fragments (blue). PC – phosphatidylcholine, DAG – diacylglycerol, SM – sphingomyelin, PE – phosphatidylethanolamine, PS – phosphatidylserine, PI – phosphatidylinositol. Intensities in different ion images do not reflect the relative abundances of the corresponding analytes.

The distribution of endogenous lipids within different skin layers was mapped at various length scales across the skin cross sections. Figure 1 displays ToF-SIMS data from an analysis covering the entire thickness of the skin, from the SC at the top to hypodermis at the lower edge of the cross section. The mass spectra show peaks from a range of specific lipids that are frequently observed also in other types of tissue, including phospholipids (phosphatidylcholine (PC), phosphatidylethanolamine (PE), sphingomyelin (SM), phosphatidylserine (PS), phosphatidylinositol (PI) and phosphatidic acid (PA)), cholesterol and diacylglycerols (DAG), see Fig. 1a–b and Supplementary Table S1. The phospholipids are all relatively homogeneously distributed over the entire cross section, suggesting that these lipids represent cell membrane components throughout dermis and viable epidermis (Fig. 1c). Cholesterol (not shown) and typical protein fragments (Fig. 1c) are also homogeneously distributed across the skin cross section, suggesting the same origin, although the protein signals may also be generated from collagen fibers, which could be observed by SEM throughout dermis (Supplementary Fig. S1). The DAG ions are assumed to represent triacylglycerol (TAG), since it is known that TAG is effectively fragmented in ToF-SIMS, producing mainly DAG ions and only low yields of intact TAG ions^34,44^. The DAG image (Fig. 1c) shows strong localization to three parts of the skin cross section; (i) the upper edge of the cross section (at the skin surface), (ii) the bottom edge of the cross section (border to the hypodermis) and (iii) specific structures deep into dermis (further discussed in the next section). The fatty acid ions C16 and C18 (16 and 18 carbon atoms, respectively) are primarily fragments of phospholipids and TAG and should not be considered to represent free fatty acids.

In addition to the compounds mainly observed in dermis, several lipids were found with strong localisation to the upper part of the skin cross section, i.e., epidermis, such as the saturated long-chain fatty acids (C24:0 and C26:0) and cholesterol sulfate (Fig. 1c). Whereas cholesterol sulfate shows appreciable signal in the upper ≈200 µm of the cross section, the C24:0 and C26:0 fatty acids are present only at the very top edge, consistent with the known localization of these fatty acids to SC.

**Figure 2:**
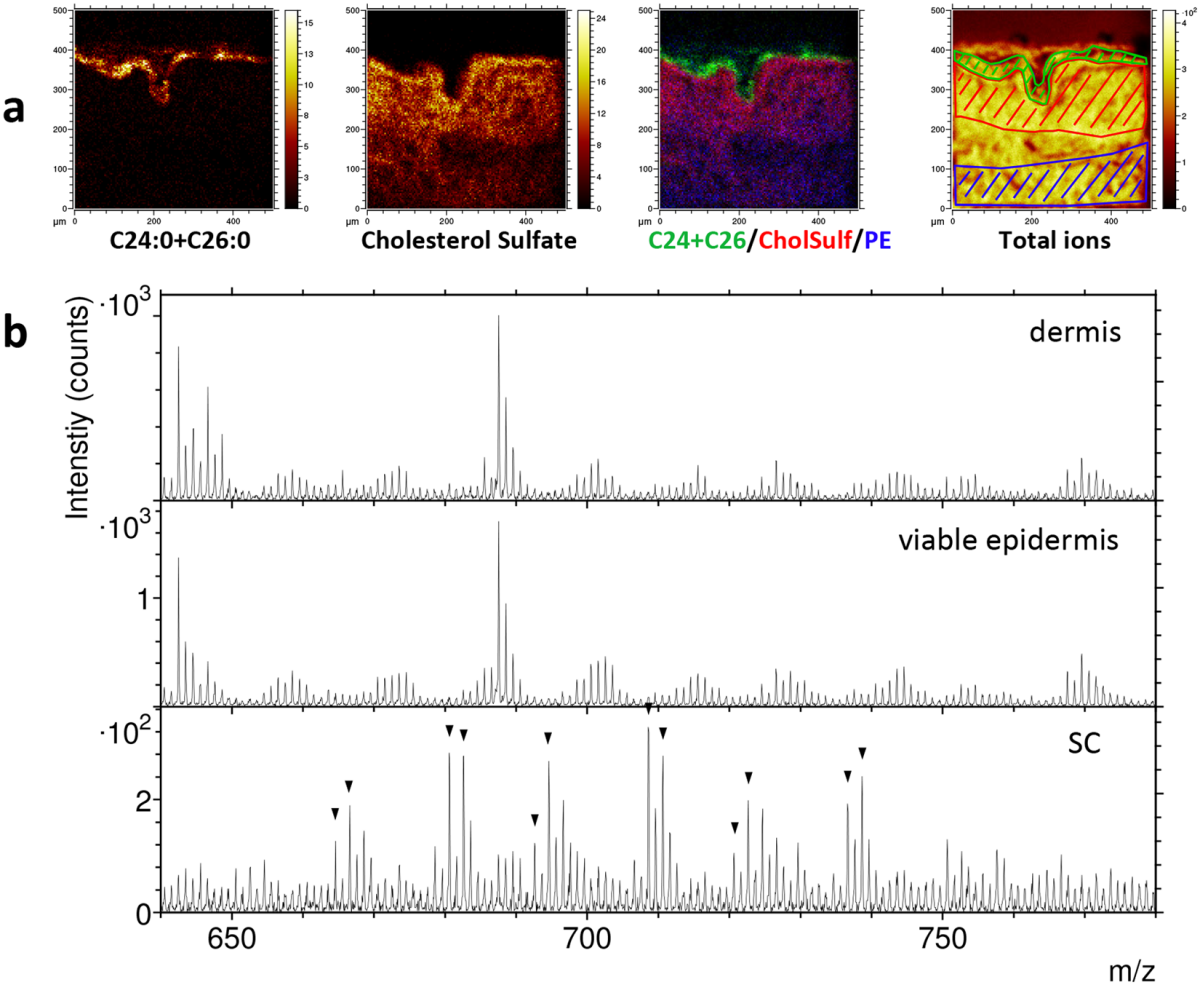
Negative ion ToF-SIMS data of the epidermal region of a skin cross section. (**a**) Ion images of (from left) C24:0 + C26:0 (m/z 367 + 395), cholesterol sulfate (m/z 465), a 3-colour overlay image of these two, also including PE fragments (m/z 140 + 180, in blue), and a total ion image. Field of view 500 × 500 µm^2^. (**b**) Spectra from regions of interest (ROIs) indicated in the total ion image in (**a**), representing dermis (blue), viable epidermis (red) and stratum corneum (green). The spectrum of SC shows a number of peaks that can be assigned to endogenous ceramides (marked by triangles and specified in Table 1). The peaks at m/z 687–688 (dermis and viable epidermis) correspond to SM16:0. Intensities in different ion images do not reflect the relative abundances of the corresponding analytes.

**Figure 3:**
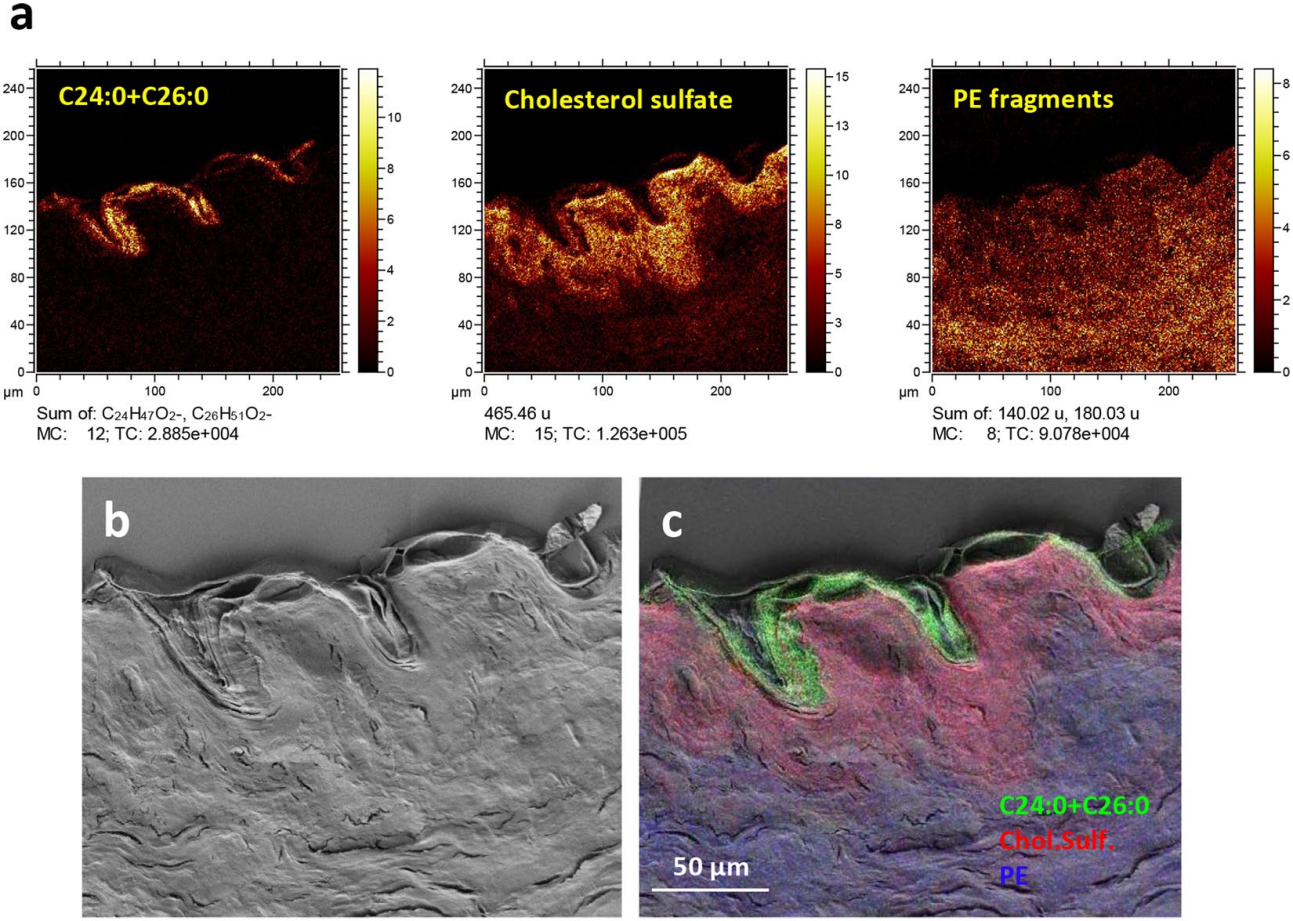
High-resolution ToF-SIMS and SEM images of the epidermal region of a skin cross section. (**a**) Negative ion images of (from left) C24:0 + C26:0 (m/z 367 + 395), cholesterol sulfate (m/z 465), and PE fragments (m/z 140 + 180). (**b**) SEM image of the same area and (**c**) SEM image with a superimposed 3-colour ToF-SIMS overlay image of C24:0 + C26:0 (green), cholesterol sulfate (red) and PE fragments (blue). Intensities in different ion images do not reflect the relative abundances of the corresponding analytes.

Analyses focusing on the epidermis region of the skin cross section confirm the exclusive localization of the C24:0 and C26:0 fatty acids to SC and cholesterol sulfate mainly to the epidermal region just below SC, i.e. the viable epidermis (Figs 2 and 3). Furthermore, negative ion mass spectra generated exclusively from specific regions of the cross section, corresponding approximately to SC, viable epidermis and dermis, respectively (indicated in the total ion image of Fig. 2a), demonstrate a very different lipid composition of SC compared to the other two skin regions (Fig. 2b, Supplementary Fig. S2). In the mass range m/z 650–750, a number of peaks are observed in the SC spectrum that can be identified as molecular ions of some of the most abundant ceramides in SC (Fig. 2b and Table 1)^6,7^. The different subclasses of ceramides in SC are characterized by 4 different types of sphingoid bases and 3 variants of the fatty acid groups, involving hydroxylation or esterification^6,7,45^, adding up to 12 different combinations that make up the different subclasses of the SC ceramides. The most abundant of these subclasses are NP, NH, AP and AH, where N and A refers to non-hydroxylated and hydroxylated fatty acid groups, respectively, and P and H refers to two different sphingoid bases, phytosphingosine and 4-hydroxysphingosine, respectively. Furthermore, the most abundant fatty acid chain lengths have been reported to be C24 and C26, with only small contributions from C16 and C18^2,3,9^, and C18 being the most abundant chain length of the sphingoid base^45^. The peaks observed in the SC mass spectrum (Fig. 2b) are consistent with NP, NH, AP and AH ceramides, each with added chain lengths of the acyl group and sphingosine base of C42, C44 and C46, e.g., corresponding to C18:0 in the sphingosine base and C24:0, C26:0 and C28:0 in the fatty acyl chain. However, since the P and H sphingosine base groups only differs stoichiometrically by one double bond, it is not possible to distinguish NH (or AH) with saturated carbon chains from NP (or AP) with one double bond in the carbon chains.

**Table 1:** Identified ions of endogenous ceramides in ToF-SIMS spectra of the stratum corneum in human skin cross sections and molecular structures of the assigned ceramides (after^6^).

Nominal mass (m/z)	Chemical formula	Molecular assignment	Observed mass (m/z)	Theoretical mass (m/z)	
**Negative ions**					
664	C_42_H_82_NO_4_^−^	NH(42:0)/NP(42:1)	664.610	664.624	
666	C_42_H_84_NO_4_^−^	NP(42:0)	666.618	666.640	
680	C_42_H_82_NO_5_^−^	AH(42:0)/AP(42:1)	680.634	680.619	
682	C_42_H_84_NO_5_^−^	AP(42:0)	682.648	682.634	
692	C_44_H_86_NO_4_^−^	NH(44:0)/NP(44:1)	692.643	692.655	
694	C_44_H_88_NO_4_^−^	NP(44:0)	694.638	694.671	
708	C_44_H_86_NO_5_^−^	AH(44:0)/AP(44:1)	708.662	708.650	
710	C_44_H_88_NO_5_^−^	AP(44:0)	710.665	710.666	
720	C_46_H_90_NO_4_^−^	NH(46:0)/NP(46:1)	720.646	720.686	
722	C_46_H_92_NO_4_^−^	NP(46:0)	722.677	722.702	
736	C_46_H_90_NO_5_^−^	AH(46:0)/AP(46:1)	736.681	736.681	
738	C_46_H_92_NO_5_^−^	AP(46:0)	738.711	738.697	
**Positive ions**					
688	C_42_H_83_NO_4_ Na^+^	NH(42:0)/NP(42:1)	688.558	688.621	
690	C_42_H_85_NO_4_ Na^+^	NP(42:0)	690.605	690.637	
704	C_42_H_83_NO_5_ Na^+^	AH(42:0)/AP(42:1)	704.632	704.616	
706	C_42_H_85_NO_5_ Na^+^	AP(42:0)	706.672	706.632	
716	C_44_H_87_NO_4_ Na^+^	NH(44:0)/NP(44:1)	716.660	716.653	
718	C_44_H_89_NO_4_ Na^+^	NP(44:0)	718.660	718.668	
732	C_44_H_87_NO_5_ Na^+^	AH(44:0)/AP(44:1)	732.660	732.648	
734	C_44_H_89_NO_5_ Na^+^	AP(44:0)	734.625	734.663	
744	\C_46_H_91_NO_4_ Na^+^	NH(46:0)/NP(46:1)	744.669	744.684	
746	C_46_H_93_NO_4_ Na^+^	NP(46:0)	746.692	746.700	
760	C_46_H_91_NO_5_ Na^+^	AH(46:0)/AP(46:1)	760.679	760.679	
762	C_46_H_93_NO_5_ Na^+^	AP(46:0)	762.681	762.695	

In the lower mass range, m/z 320–430, the negative ion ToF-SIMS spectra reveal the presence of several long-chain fatty acids in SC, from C21:0 to C28:0 (Supplementary Fig. S2, Table S1). The most abundant ones are C24:0 and C26:0, in agreement with previous studies^7,9,45^, but interestingly, significant signal is observed for all chain lengths between C21 and C28, including those with odd numbers of carbon atoms, i.e., C21:0, C23:0, C25:0 and C27:0, consistent with previous observations of odd carbon fatty acyl chains in human SC ceramides^7,45^. It should be emphasized that both free fatty acids and fatty acids bound to lipids (including ceramides) are expected to contribute to the ToF-SIMS signal.

The localizations of the SC lipid components in the skin cross section are further investigated in ToF-SIMS images obtained with the instrument optimized for high lateral resolution in combination with SEM (Fig. 3, Supplementary Fig. S3). The ToF-SIMS images and the overlay with the SEM image clearly shows that the long-chain fatty acids (C24:0 + C26:0) are exclusively located to the layered structures in the SEM image that correspond to SC (Fig. 3), and that cholesterol sulfate is mainly located to the region just below SC, corresponding to the viable epidermis, in good agreement with previous reports^13,14^. The PE fragment signal shows an increasing intensity towards the deeper layers (Fig. 3a,c), consistent with the interpretation that these ions represent phospholipids, which are mainly present in viable cell membranes. Additional ion images show clear localization to SC of the molecular ceramide peaks listed in Table 1 and the odd carbon fatty acids (Supplementary Fig. S3). Cholesterol shows some localization to SC, although the signal is appreciable over the entire cross section area, consistent with its known presence in SC but also in the viable cell membranes. An interesting ion image was obtained for the phosphate ion, PO_2_^−^, in which aggregates around 5 µm in diameter are observed in the viable epidermis (Supplementary Fig. S3). The origin of these aggregates is, however, unknown. Chlorine ions display an enhanced signal from the entire epidermis, including SC, and considerably lower signal in dermis (Supplementary Fig. S3).

### Penetration of topically applied oregano essential oil in skin cross sections

The distribution of oregano essential oil (EO) in the skin was measured by ToF-SIMS and GC-MS after topical application using a Franz cell. For both analysis techniques, the EO was monitored by detection of carvacrol, which is the main component of oregano EO (about 56%).

**Figure 4:**
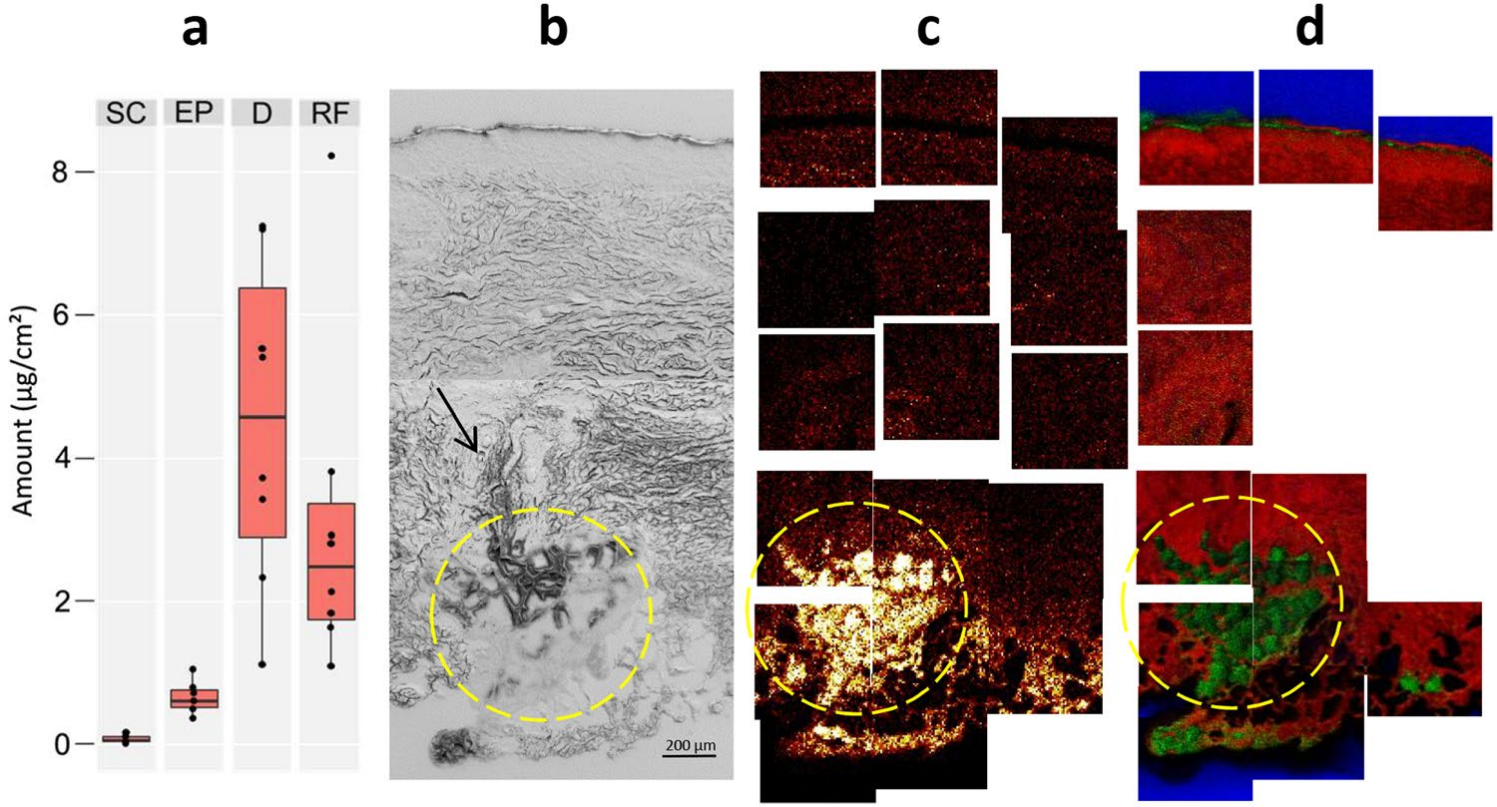
Localization of topically applied carvacrol in skin cross section. (**a**) GC-MS analysis of the carvacrol concentrations in different skin layers (SC – stratum corneum, EP – viable epidermis, D – dermis, and RF – receptor fluid). See also Supplementary Table S2. (**b**) Large-area SEM image (stitched) of the skin cross section (the arrow indicates the position of a possible hair follicle, see also Supplementary Fig. S1), (**c**) negative ToF-SIMS images of carvacrol (m/z 149) and (**d**) positive ion 3-colour images of DAG (m/z (575–579)+(601–605), green), representing triglycerides, PC fragments (m/z 86 + 184, red) and silicon (m/z 28, blue), acquired at the same skin cross section area as the SEM image in (**b**). The yellow circles indicate the location of a possible secretory coil of an eccrine sweat gland (see text). Intensities in different ion images do not reflect the relative abundances of the corresponding analytes.

The GC-MS analysis showed that most of the detected carvacrol was found in dermis, whereas only small amounts were detected in SC and viable epidermis (Fig. 4a and Supplementary Table S2). Significant amounts were detected in the receptor fluid of the Franz cell, indicating that some carvacrol penetrated through the entire thickness of the skin sample. Furthermore, only 25–30% of the applied carvacrol was accounted for by the detected quantities. Despite occlusion of the donor compartment with aluminium foil during exposure, most of the applied carvacrol was thus evaporated from the skin surface during application and analysis, due to its high volatility.

The ToF-SIMS analysis was performed by acquiring data from numerous analysis areas covering most of the skin thickness of each cross section, from SC to the border to the hypodermis (Fig. 4b–d). Carvacrol was monitored by the molecular peak, (M-H)^−^, in negative ion mode at m/z 149.10. The results show a low but significant carvacrol signal throughout the entire cross section (Supplementary Fig. S4). No accumulation was observed at the skin surface, indicating a relatively fast penetration of EO through the skin surface, in agreement with the GC-MS results.

For some samples, very strong carvacrol signals were observed at certain structures in the deeper parts of dermis (Fig. 4c). Comparing the carvacrol distribution with other ion images revealed that these carvacrol-rich structures are closely correlated with structures of high signal intensities from DAG ions (Fig. 4d), suggesting a preferential location of carvacrol to fat-rich structures in dermis. The appearance, location and high triglyceride content of these structures indicate that they correspond to adipocytes, possibly associated with the secretory coil of an eccrine sweat gland. However, although SEM images of the same area indicate the presence of a hair follicle (Fig. 4b arrow, Supplementary Fig. S1), the association of the fat-rich structures to a sebaceous gland is less likely, due to the expected location of sebaceous glands to the upper parts of dermis.

### Penetration of topically applied ceramide in skin cross section

**Figure 5:**
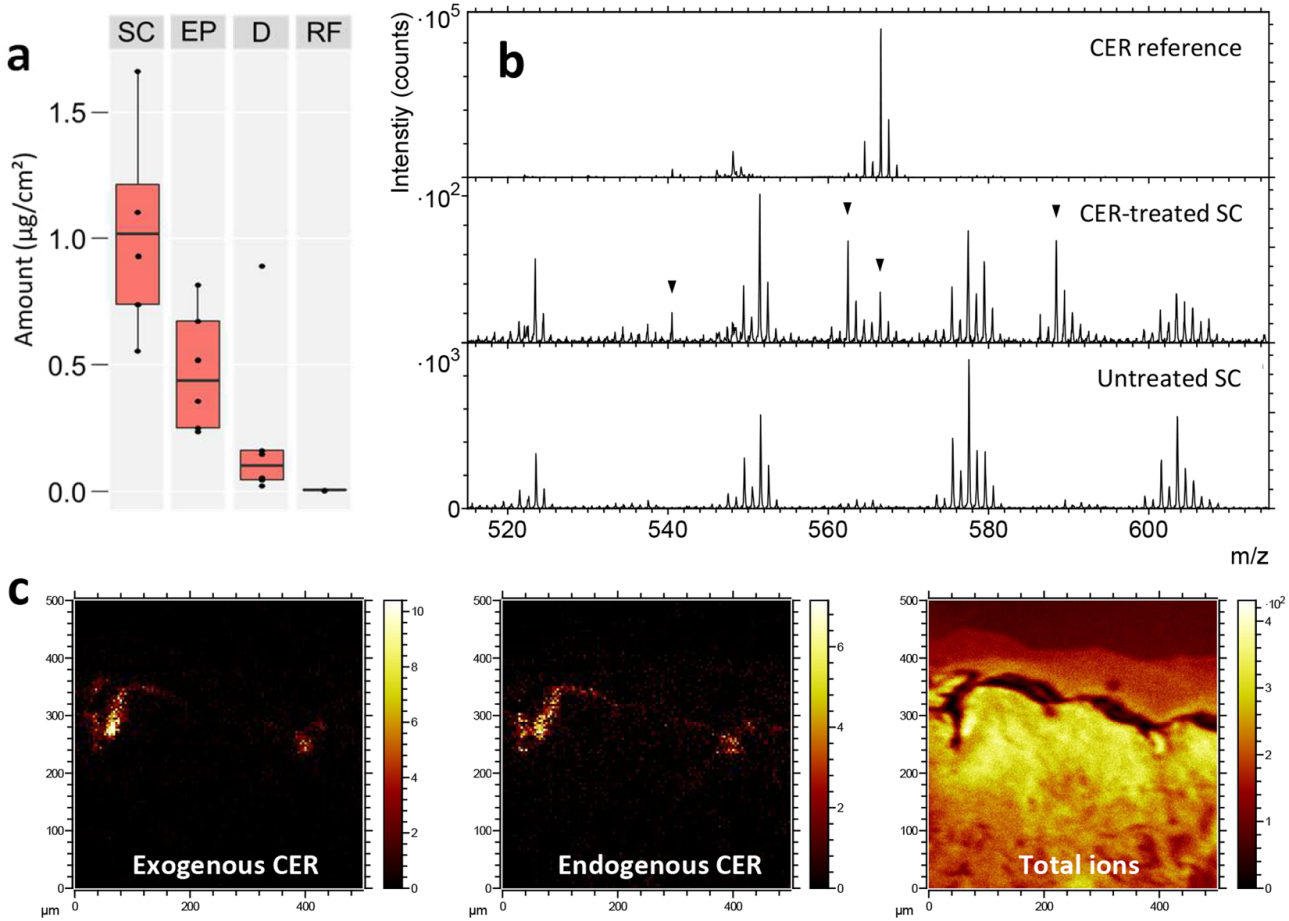
Detection and localization of topically applied ceramide in skin cross sections. (**a**) LC-MS analysis of the ceramide concentration in different skin layers (SC – stratum corneum, EP – viable epidermis, D – dermis, and RF – receptor fluid). See also Supplementary Table S2. (**b**) Positive ToF-SIMS spectra in the mass range of the ceramide molecular ion for a ceramide reference sample (top), the SC region of a skin sample treated with a ceramide-containing formulation (center, arrows indicate peaks assigned to ceramide), and the SC region of an untreated skin sample (bottom). (**c**) Ion images of the epidermal region of a skin sample treated with a ceramide-containing formulation, showing (from left); exogenous ceramide (m/z 562 + 566 + 588), endogenous ceramide (m/z 690 + 704 + 716 + 718 + 732), and total ions. Field of view 500 × 500 µm^2^. Intensities in different ion images do not reflect the relative abundances of the corresponding analytes.

As for oregano essential oil (EO), the distribution of an exogenous ceramide (CerNS18 or Cer(d18:1/18:0)) was measured both by ToF-SIMS and cutaneous absorption analysis (now LC-MS), after topical application using a Franz cell. Using a validated LC-MS-MS method, the exogenous ceramide was mostly found in SC and in epidermis, with lower amounts detected in dermis (Fig. 5a and Supplementary Table S2). Ceramide was undetected in the receptor fluid. This distribution profile is consistent with the lipophilic properties of ceramide, which causes this chemical to be retained in the upper layer of the skin.

The penetration of ceramide into human skin was also investigated by mapping the ceramide distribution in skin cross sections using ToF-SIMS. The exogenous ceramide in the topical formulation was distinguished from the endogenous ceramides in SC by their molecular weights. Whereas the ceramide contained in the topical formulation was CerNS18, with a molecular weight of 565 Da, the endogenous SC ceramides are in the 650–750 Da mass range (as demonstrated above), thus, allowing for unambiguous and independent detection of the two types of ceramides, given that they are monitored using the intact, or nearly intact, molecular ions.

**Figure 6:**
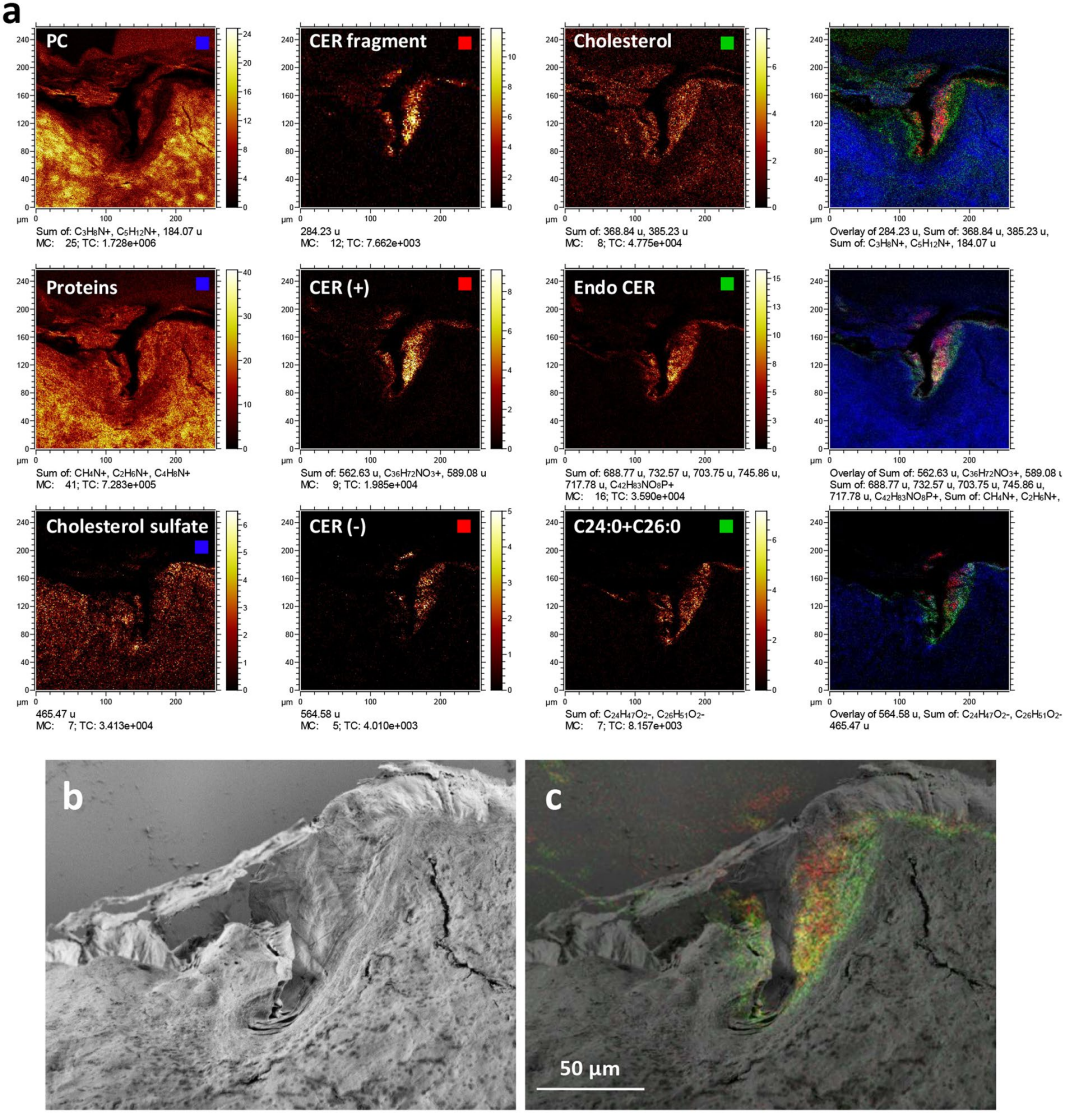
High resolution ToF-SIMS and SEM images of the SC region of a cross section skin sample treated with a ceramide-containing formulation. (**a**) Ion images representing (in each row) viable epidermis components, exogenous ceramide and endogenous SC lipids. The right images are 3-colour overlays of the left three images in the same row. Top row (from left, positive ions): PC fragments (m/z 58 + 86 + 184, blue in 3-colour overlay), ceramide fragment (m/z 284, red), cholesterol (m/z 369 + 385, green). Center row (from left, positive ions): protein fragments (m/z 30 + 44 + 70, blue), exogenous ceramide (m/z 562 + 566 + 588, red), endogenous ceramide (m/z 688 + 704 + 718 + 732 + 746 + 760, green). Bottom row (from left, negative ions): cholesterol sulfate (m/z 465, blue), exogenous ceramide (m/z 564, red), C24:0 + C26:0 (m/z 367 + 395, green). Field of view: 250 × 250 µm^2^. (**b**) SEM image of the same area, (**c**) SEM image with superimposed ToF-SIMS overlay image of exogenous ceramide (red) and endogenous ceramide (green). Intensities in different ion images do not reflect the relative abundances of the corresponding analytes.

The ToF-SIMS data from the skin cross sections revealed a clear localization of the exogenous ceramide to SC, but no ceramide could be detected at the deeper layers. It was further observed that CerNS18 was preferentially detected in locations corresponding to furrows in the skin, where SC bends slightly into the deeper layers of epidermis (Figs 5 and 6, Supplementary Figs S5–S7), as shown previously^21^. In the positive ion mode (Fig. 5b,c), the exogenous ceramide was detected not only by the protonated molecular ion (m/z 566.56), which is the main peak of the pure ceramide reference spectrum in the mass range of the intact ceramide molecule, but also by two peaks at m/z 588.53 and 562.52, which can be assigned to sodium complexes of the intact ceramide molecule and of the ceramide molecule after loss of a minor fragment, respectively (Fig. 5b). The latter assignment is motivated by the presence of the corresponding protonated ion at m/z 540.56 in, both, the skin cross section and ceramide reference spectra. The assignment of all these peaks to the exogenous ceramide is confirmed by their absence in spectra of the SC region in cross section samples of skin that has not been treated with the ceramide-containing formulation (Fig. 5b). In addition, the endogenous SC ceramides could be detected with relatively strong signal and clear localization to SC by their sodium molecular complex ions (Fig. 5c, Table 1), thus allowing for comparison of the endogenous ceramide composition (and distribution) in the treated skin sample with that of the untreated skin. In the present case, however, no significant difference could be observed between the untreated and ceramide-treated skin samples with respect to the relative peak intensities of the endogenous ceramides (Supplementary Fig. S8).

In the negative ion mode, exogenous ceramide was detected by the deprotonated molecular ion (m/z 564.50), which is also the dominating peak in the ceramide reference spectrum in this mass range (Supplementary Fig. S5). Also in this case, the exogenous ceramide signal was exclusively located to SC, and the untreated skin sample showed no significant signal at this peak.

The spatial distribution of the exogenous ceramide was investigated at high image resolution, in order to allow for detailed comparison with the distributions of the endogenous SC lipids (Fig. 6). Figure 6a shows three sets of ion images, in which each row includes images that represent (from left) molecular components of the viable epidermis, exogenous ceramide, endogenous SC lipids, and a 3-colour overlay image of these three images, respectively. Detailed inspection of the images shows that the three images representing SC lipids (cholesterol, ceramides and C24:0 + C26:0 fatty acids, respectively) share a similar lateral distribution. Furthermore, the three images representing exogenous ceramide also display similar distributions. However, there is a small but consistent difference between the distributions of the exogenous ceramide and the SC lipids, in that most of the exogenous ceramide appears to be located slightly closer to the skin surface, whereas the signal at the deeper parts of SC are considerably lower (particularly evident in the 3-colour overlay images of Fig. 6a). The different distributions of the exogenous ceramide and the SC lipids (green) thus indicate that the exogenous ceramide is not homogeneously distributed in SC, but rather exhibits a gradient with lower concentrations towards the bottom layers of SC.

SEM analysis of the same area, and an overlay of the SEM and ToF-SIMS images (Fig. 6b,c), provide additional information about the location of the exogenous ceramide. The SEM image (Fig. 6b) shows that the analysis area corresponds to a skin cross section region where SC has been folded into the skin in a wrinkle structure and that the SC regions exposed for analysis display various characteristics: whereas parts of the exposed SC correspond to the characteristic layered structure of the SC cross section, a relatively large region appears to be a flattened part of the top SC surface. Furthermore, the ToF-SIMS overlay with the SEM image (Fig. 6c) shows that the exogenous ceramide is mainly located to the SC region corresponding to the top SC surface, whereas the endogenous SC lipids are located mainly to the layered cross section region of SC, although both the exogenous and endogenous components produce significant signals in both regions. The observation that the exogeneous ceramide is mainly located to the top SC surface and thus not homogeneously distributed in SC indicates that ceramide does not easily penetrate into SC. Additional ToF-SIMS/SEM overlay images supporting this conclusion is provided in Supplementary Figs S6 and S7. These results are in good agreement with previous studies showing that the penetration of topically applied ceramides is limited to the superficial layers of SC, except in the presence of penetration enhancers or for very short carbon chains in the fatty acyl groups^16,21,46^.

## Discussion and Conclusions

In this work, the capabilities of a combined approach were demonstrated, using ToF-SIMS imaging together with cutaneous absorption quantification and SEM structural characterization. This approach provided information about the penetration and final localisation of topically applied compounds in *ex vivo* human skin, as well as the spatial and compositional distributions of some of the most abundant endogenous skin lipids. Spatial distributions consistent with prior knowledge were obtained for long-chain fatty acids and ceramides (stratum corneum) and for cholesterol sulfate (viable epidermis). Carvacrol (the main component of oregano essential oil) was found to effectively penetrate SC, but also to accumulate in fat-rich structures deep in dermis, such as residual adipocytes from hypodermis and possibly eccrine sweat glands. In contrast, topically applied ceramide was found by ToF-SIMS imaging to be exclusively located to SC, with an increased abundance at the upper layers of SC, whereas cutaneous absorption analysis showed the highest amounts in SC, but also significant quantities in the viable epidermis and dermis.

The results show that ToF-SIMS can provide images of endogenous and exogenous compounds in skin cross sections with high specificity (compared to, e.g., optical microscopy and FTIR/Raman imaging) and at spatial resolutions down to the micrometer range. The use of skin cross sections allows for acquisition of overview images of large areas, followed by immediate detailed investigation of areas or structures of interest at different depths of the skin. Furthermore, subsequent analysis of the same areas/structures by SEM makes it possible to identify skin structures that may be related to features of the molecular images obtained by ToF-SIMS. While the chemical specificity and spatial resolution are the main advantages of ToF-SIMS, cutaneous absorption analysis provides better sensitivity and quantification capabilities than ToF-SIMS and is therefore an important complementary technique.

For the carvacrol penetration study, a good agreement was observed between the GC-MS quantification and ToF-SIMS imaging, in both cases showing strong location to the dermis. 84% ± 6.6 of the total amount of carvacrol in the skin was detected in dermis by GC-MS. However, ToF-SIMS imaging clearly showed that carvacrol was not homogenously distributed in dermis, as a clear colocalisation with structures dominated by triglycerides (and DAG) was observed. Although the identity of these structures cannot be conclusively assigned, their appearance and location deep in dermis may indicate that some of them are associated with the secretory coil of an eccrine sweat gland. However, carvacrol accumulation is also found for triglyceride-containing structures at the border to hypodermis, likely to be residual adipocytes. The effective penetration and strong affinity to triglyceride is consistent with the low molecular weight of carvacrol (150 Da) and its strong lipophilic/hydrophobic properties.

Upon ToF-SIMS imaging, exogenous ceramide was detected only in SC, and there was even a gradient showing lower concentration in the deeper SC layers (demonstrated by differences compared to the distributions of endogenous SC lipids), which is also consistent with previous studies^21,46^. In contrast, cutaneous absorption analysis showed significant amounts of ceramide in viable epidermis. This apparent contradiction may be related to the different sensitivities of the two methods. First, LC-MS showed considerably higher amounts of exogenous ceramide in SC compared to viable epidermis, suggesting that the amount in viable epidermis may be below the detection limit for ToF-SIMS. Second, the epidermal volume is about 10 times larger than the SC volume, indicating that the ceramide detected by LC-MS in viable epidermis may be up to tenfold diluted, which further reduces the concentration available for detection by ToF-SIMS. However, it has previously been pointed out^47^ that a potential problem of using tape stripping for sampling SC layers is that parts of the SC may not be completely removed, particularly in damaged regions or furrows in the skin, thus leaving some parts of the SC to be included in the analysis of the deeper skin layers. Since the ToF-SIMS images show that the exogeneous ceramide is preferentially located to skin furrows, we cannot exclude the possibility that the ceramide detected in viable epidermis in the cutaneous absorption analysis corresponds to such residues of SC. It is clear that further studies are needed to characterize the extent of ceramide penetration into and across SC.

An important advantage of the presented approach using skin cross sections is the possibility to access the entire depth of the skin for detailed analysis of specific structures, and the clarity of the results that this provides with respect to the identity and location of specific molecular species in the skin. However, an important disadvantage is that only skin explants can be analysed, as opposed to fresh or viable skin. Also, the preparation of the cross-section samples is critical and can be problematic, especially considering that embedding of the skin sample should be avoided due to the added data complexity related to the strong organic signals generated by the embedding medium and the compromising effect that the embedding process may have on the lipid localisation. A final disadvantage of using cross section samples is that the sensitivity is probably lower than that of direct analysis of the skin surface, e.g., through analysis of successive tape strips^43^. However, it is likely that the sensitivity can be improved for the analysis of cross sections by the use of argon cluster ion sputtering, which would increase the sample volume (i.e., depth) available for analysis.

## Methods

### Skin samples and topical formulations

Normal abdominal human skin from anonymous healthy female donors was obtained during plastic surgery procedures according to the French regulations (article L. 1243-4 of the French public Health Code) and Declaration of Helsinki act. Patients’ written informed consents were collected and kept by the surgeon. Only age, sex and anatomical site of samples were specified to the authors. The authors did not participate in sample collection.

After collection at surgery, full thickness *ex vivo* human skin samples were frozen at −20 °C and then stored for less than 6 months before preparation for analysis. After thawing, the skin samples were checked visually, where those having stretchmarks, holes, damage, etc. were discarded prior to punching. All skin samples were then gently cleaned with pure water and remaining subcutaneous fat was removed. The thickness of each skin sample was measured using a micrometer and the mean thickness ± sd was determined to 1248 ± 315 µm and 2749 ± 490 for the skin samples used in the ceramide and carvacrol study, respectively. Full thickness *ex vivo* human skin was used instead of split thickness skin, as split thickness skin is much more difficult to handle and the skin consumption is greater. A list of all samples included in the study, and the analyses conducted on each of them, is provided in Supplementary Table S3, with references to the data presented in figures and tables.

Studies on the penetration of carvacrol and ceramide were performed according to OECD guidelines (OECD, 2004b. OECD guideline for the testing of chemicals. Skin absorption: *in vitro* method. 428. Adopted 13 April, 2004). The skin samples were mounted on static Franz cells having 2 cm² exposure areas. The receptor chamber was filled with an aqueous solution of 0.9% (w/v) NaCl and 0.25% (w/w) Tween 80. This surfactant was added in the receptor fluid (RF) to guarantee sufficient solubility of the studied chemicals in the receptor fluid. A circulating bath was used to maintain a constant temperature of each cell. The RF was stirred during the entire experiment to ensure a good homogeneity. The skin was equilibrated for one hour before formula application to guarantee reproducible skin hydration and skin temperature. The skin temperature was measured using a digital contact probe and remained at 32 ± 1 °C. The skin integrity was checked using trans-epidermal water loss (TEWL, Tewameter TM 300, Monaderm, Monaco) and skin samples having TEWL values higher than 10 g/(cm^2^h) were rejected. This cut off TEWL value was based on historical data.

Green Oregano essential oil (EO) and Ceramide were formulated at 1% (w/w) in a gel and at 1% (w/w) in emulsion, respectively, see Supplementary Table S4 for complete compositions. The formulations were applied homogeneously onto the skin at 5 mg/cm² using a spatula. The spatula was weighed before and after application to determine the exact amount of formulation applied on the skin. For the application of Green Oregano EO, the skin was covered with aluminium foil to limit carvacrol evaporation.

At the end of the exposure time (16 h), the skin surface was washed with Lauryl Ether Sulfate (LES) surfactant solution in water (3.5% (w/v)), rinsed with water and dried with cotton buds. All washing solutions were collected in glass scintillation vials and subsequently analysed.

Carvacrol (>98%) [499-75-2] was obtained from TCI Europe NV (Zwijndrecht, Belgium). N-stearoyl-D-erythro-sphingosine (CerNS18 ceramide) [54422-45-6], Green Oregano essential oil [91721-63-0] and Lauryl Ether Sulfate (LES) [3088-31-1] solution at 70% were obtained from L’Oréal laboratories (Aulnay-sous-Bois, France). Tween® 80 [9005-65-6] was obtained from Sigma-Aldrich (Lyon, France).

All reagents used (receptor fluid, chromatography and sample extraction) were of analytical grade: sodium chloride [7647-14-5], methanol (MeOH) [67-56-1], acetonitrile (ACN), Tert-Butyl Methyl Ether (TBME) [1634-04-4], formic acid (FA) [64-18-6] and ammonium acetate [631-61-8]. Ultra-high quality water was obtained from a Milli-Q® (Millipore, Bedford, MA, USA) system or from Carlo Erba (Val De Reuil, France).

The formulations containing carvacrol and ceramide were provided by L’Oréal laboratories (Aulnay-sous-Bois, France).

### Cutaneous absorption: skin layer sampling

For the cutaneous absorption studies, the washing and receptor fluids were collected for analysis. Tape Stripping was performed using standardized adhesive tapes (D-Squame™, CuDerm Corporation, Dallas, TX, USA). A maximum of 20 strips were performed on each skin sample to remove the major part of SC. Strips were pooled in groups of 10 tapes in a scintillation vial prior to extraction). After tape stripping, viable epidermis was removed from dermis by heating the area of interest for 30 seconds to 1 min with a hair dryer, leading to a temperature of the skin surface of 60 °C measured with an infrared probe. The viable epidermis was then gently removed from dermis. Both viable epidermis and dermis were placed into individual scintillation vials.

### Cutaneous absorption: Sample preparation and analytical method

The dedicated analytical method was validated for each chemical (e.g. carvacrol and ceramide), according to previously defined criteria^48^. These criteria are classically used for validation of bioanalytical method^49^.

The specificity of the analytical method was controlled for each blank matrix (e.g. strips, viable epidermis, dermis and receptor fluid). Linearity was determined between the LLOQ and ULOQ ng/ml, with an accuracy below ± 15%, except at the LLOQ, which was below ± 20%. Accuracy and precision was determined at least at two QC theoretical concentrations: low and middle. All QCs remained within the acceptance criteria (accuracy% <  ± 15%). QC were used to control matrix effect and recovery extraction.

#### EO Green Oregano

TBME was used as solvent extraction. For strips, viable epidermis and dermis, 3, 1.5 and 2.5 ml were used, respectively. Viable epidermis and dermis were extracted with back and force agitation for 21 hours whereas strips were extracted for 1 hours only. 1 ml receptor fluid and washing were extracted with 2 ml TBME and shaked for 1 hour. All samples were stored at −80 °C before analysis.

All samples were analyzed onto a GC/MS QP-2010 Ultra system (Shimadzu, Kyoto, Japan). The analytical system was managed by the software GC/MS Solutions.

The analytical column used was a HP1 MS (Agilent, Santa Clara, CA, USA) (0.2 mm × 50 m × 0.33 µm). The carrier gas was helium with a flow rate of 1.28 ml/min. The initial column temperature of 80 °C increases to 260 °C within 12 min and then held for 5 min. Injections (5 µl) were made in a split mode with ratio 20. Injection temperature was set at 250 °C. Ionization mode used was Electron Impact. Single Ion Monitoring (SIM) was used for quantification with ion at m/z 135. Ion at m/z 150 and m/z 91 were used for confirmation.

LLOQ and ULOQ were at 5 and 1250 ng/ml, respectively. QCs used for extraction recovery and matrix effect was set at 22 and 168 ng/ml at least in triplicate. Extraction recovery for each analyzed compartment is reported in Supplementary Table S5.

#### Ceramide

Methanol (MeOH) was used as extracting solvent. For washing, strips, viable epidermis and dermis, 17, 3, 2 and 5 ml were used, respectively. Viable epidermis and dermis were extracted with back and forth agitation for 3 hours. Washing extract and receptor fluid was diluted tenfold and twofold with MeOH, respectively. All samples were filtered on Millex filter 0.45 µm (Merck Millipore, Burlington, MA, USA) before analysis.

All samples were analysed onto an LC/MS-MS system (Agilent HP1200 LC system coupled with a mass spectrometer API 3200 (Sciex, Concord, Ontario, Canada). The analytical system was managed by the software Analyst version 1.6.

The analytical column used was a Luna C8 from Phenomenex (Torrance, CA, USA) (30 × 2.1 mm, 5 µm) and analysis were carried out with a gradient elution with mobile phases of 0.1% formic acid (FA) in water (A) and MeOH (B). The column temperature was fixed at 50 °C, the volume of the injection was 10 µl and the flow rate at 0.8 ml/min. Ionization mode used was APCI positive. Multiple Reaction Monitoring (MRM) was used for detection with the following transition 566.6 → 266.4.

LLOQ and ULOQ were at 2 and 2500 ng/ml, respectively. QCs used for extraction recovery and matrix effect was set at 20 and 500 ng/ml at least in triplicate. Extraction recovery for each analyzed compartment is reported in Supplementary Table S6.

### ToF-SIMS analysis

The skin cross section samples were prepared at Histocenter AB (Västra Frölunda, Sweden) by cutting ca 10 µm thick slices of the skin samples (without embedding medium) using a cryosectioning device at −20 °C, placing them on a slightly warmed-up silicon substrate (for attachment) and then quickly freezing the substrate again to −20 °C. After cryosectioning, the cross section samples were stored at −80 °C until analysis (<10 days).

The ToF-SIMS analyses were conducted at a sample temperature of −80 °C, except for the samples used for investigation of ceramide penetration, which were analyzed at room temperature. For analysis at −80 °C, the frozen cross section samples were mounted on the precooled sample holder inside a container partly filled with liquid nitrogen. The sample holder was then quickly transferred to the vacuum chamber of the ToF-SIMS instrument, equipped with liquid nitrogen cooling and temperature control facilities, making sure that the sample temperature never increased above −80 °C prior to ToF-SIMS analysis. The samples for analysis at room temperature were quickly thawed in a dry atmosphere before they were mounted on the sample holder and transferred to the ToF-SIMS instrument. The oregano essential oil reference sample was analyzed at −80 °C after deposition on a silicon substrate and immediate cooling by liquid nitrogen. The ceramide reference sample was analyzed at room temperature after deposition/drying of the solution on a silicon substrate.

In ToF-SIMS, a focused beam of high energy (primary) ions irradiates the sample surface, resulting in the emission of secondary ions, which are analysed in a ToF analyser to provide molecular information about the sample surface^32,33,35^. Each measurement normally involves scanning the primary ion beam over a selected analysis area and acquiring mass spectrometry data from each pixel within the analysed area. The acquired data can be displayed as mass spectra of the entire analysis area or of selected regions of interest (ROI) within the analysis area, or as ion images showing the signal intensity distribution of selected ions over the analysis area. Absolute quantification of analyte concentrations is difficult in ToF-SIMS because the measured secondary ion yields depend not only on analyte concentration but also on other factors, such as matrix effects (and internal calibration is usually not possible). In contrast, relative concentrations, comparing analyte abundances between samples or in different regions of a sample, is normally reliable if proper consideration to possible matrix effects is taken.

The ToF-SIMS analyses were conducted under static SIMS conditions in a TOFSIMS IV instrument (IONTOF GmbH, Münster, Germany), using 25 keV Bi_3_^+^ primary ions and low-energy electron flooding for charge compensation. Positive and negative ion data were acquired with the instrument optimized for high mass resolution (m/Δm ≈ 3000–6000, lateral resolution ≈ 3–5 µm) or for high lateral resolution (m/Δm ≈ 300, lateral resolution ≈ 500 nm). The identification of lipids in the acquired mass spectra were based on agreement between theoretical mass and observed peak positions in spectra obtained at high mass resolution (see Table 1 and Supplementary Table S1), previous analyses of pure compounds, published ToF-SIMS results of lipids, and on the expected lipid composition and mass spectrum data from previous studies of skin using other mass spectrometric methods (e.g., ESI-MS and MALDI-MS).

The full thickness of the skin was analysed for all samples, in most cases using the so called macro raster utility of the instrument (Fig. 1). Data from relevant parts of the skin cross section were then acquired in several analysis areas (10–15) with the instrument in the high mass resolution mode (Figs 2, 4 and 5). The analysis of ceramide penetration was thus focused on the epidermis region, whereas carvacrol penetration was studied across the entire depth of the skin. Finally, specific areas of interest were analysed at higher magnifications with the instrument optimized for high image resolution (Figs 3 and 6).

### SEM analysis

After ToF-SIMS analysis, the samples were thawed to room temperature and subsequently analyzed by scanning electron microscopy (SEM). Prior to SEM analysis, the skin cross section samples were coated with a 15 nm thick layer of Ag/Pd to prevent charging effects during analysis. The SEM analyses were conducted in a Zeiss Supra 40VP FEG-SEM instrument at 2 keV electron energy, 4–7 mm sample distance and using the SE2 secondary electron detector (Everhardt-Thornley type).

## Electronic supplementary material

Supplementary Information

## Data Availability

The datasets generated during the current study are available from the corresponding author on reasonable request.
