# Supplementary material for: Imaging the distribution of skin lipids and topically applied compounds in human skin using mass spectrometry
Source: Sci Rep. 2018 Nov 12;8:16683. doi: 10.1038/s41598-018-34286-x (PMC6232133; doi:10.1038/s41598-018-34286-x)
Supplement: Supplementary file 1 — Supplementary Information [file 41598_2018_34286_MOESM1_ESM.pdf]

Supplementary Material

Imaging the distribution of skin lipids and topically applied compounds in human skin using mass spectrometry

Peter Sjövall[1,*], Lisa Skedung[2], Sébastien Gregoire[3], Olga Biganska[4], Franck Clément[4] and Gustavo S. Luengo[3,*]

[1] RISE Research Institutes of Sweden, Chemistry and Materials, SE-50115 Borås, Sweden
[2] RISE Research Institutes of Sweden, Surface, Process and Formulation, SE-11428 Stockholm, Sweden
[3] L'OREAL Research and Innovation, 93601 Aulnay-sous-Bois, France
[4] L'OREAL Research and Innovation, 94550 Chevilly Larue, France

* Corresponding authors: peter.sjovall@ri.se, GLUENGO@rd.loreal.com

Contents:

Supplementary Table S1	Assignment of peaks observed in positive and negative ToF-SIMS spectra
Supplementary Table S2	Results from cutaneous absorption studies
Supplementary Table S3	Sample information
Supplementary Table S4	Composition of carvacrol and ceramide formulations
Supplementary Table S5	Carvacrol extraction recovery from different skin layers
Supplementary Table S6	Ceramide extraction recovery from different skin layers
Supplementary Figure S1	SEM images of different regions of skin cross section
Supplementary Figure S2	Negative ToF-SIMS spectra from SC, viable epidermis and dermis in the mass range containing fatty acid ions
Supplementary Figure S3	High-resolution ToF-SIMS images of epidermis region
Supplementary Figure S4	ToF-SIMS spectra of carvacrol peak at different locations in skin cross section
Supplementary Figure S5	Negative ToF-SIMS ion images and spectra from ceramide-treated skin
Supplementary Figure S6	High-resolution ToF-SIMS and SEM images of ceramide-treated skin
Supplementary Figure S7	High-resolution ToF-SIMS and SEM images of ceramide-treated skin
Supplementary Figure S8	Positive ToF-SIMS spectra of endogenous ceramide peaks comparing ceramide-treated and untreated skin

Supplementary Table S1 List of identified peaks in positive and negative ToF-SIMS spectra of human skin cross sections. PC – phosphatidylcholine, MAG – monoacylglycerol, DAG – diacylglycerol, Cer – ceramide, SM – sphingomyelin, PE – phosphatidylethanolamine, C – fatty acid, PI – phosphatidylinositol, PS – phosphatidylserine, PA – phosphatidic acid

Positive ions

Nominal mass (m/z)	Chemical formula	Molecular assignment	Observed mass (m/z)	Theoretical mass (m/z)
**44**	C_2H_6N	Protein fragment	44.054	44.050
**70**	C_4H_8N	Protein fragment	70.071	70.066
**86**	$C_5H_{12}N$	PC fragment	86.110	86.097
**184**	$C_5H_{15}NPO_4$	PC fragment	184.101	184.074
**206**	$C_5H_{14}NPO_4$ Na	PC fragment	206.080	206.056
**224**	$C_8H_{19}NPO_4$	PC fragment	224.116	224.105
**313**	$C_{19}H_{37}O_3$	MAG 16:0	313.269	313.274
**337**	$C_{21}H_{37}O_3$	MAG 18:2	337.274	337.274
**339**	$C_{21}H_{39}O_3$	MAG 18:1	339.290	339.290
**341**	$C_{21}H_{41}O_3$	MAG 18:0	341.300	341.306
**367**	$C_{27}H_{43}$	Cholesterol	367.331	367.336
**369**	$C_{27}H_{45}$	Cholesterol	369.358	369.352
**384**	$C_{27}H_{44}O$	Cholesterol	384.331	384.339
**385**	$C_{27}H_{45}O$	Cholesterol	385.339	385.347
**430**	$C_{29}H_{50}O_2$	Vitamin E	430.375	430.381
**547**	$C_{35}H_{63}O_4$	DAG 32:2	547.494	547.473
**549**	$C_{35}H_{65}O_4$	DAG 32:1	549.496	549.489
**551**	$C_{35}H_{67}O_4$	DAG 32:0	551.512	551.504
**566**	$C_{36}H_{72}NO_3$	CerNS18	566.558	566.551
**573**	$C_{37}H_{65}O_4$	DAG 34:1	573.481	573.488
**575**	$C_{37}H_{67}O_4$	DAG 34:2	575.511	575.504
**577**	$C_{37}H_{69}O_4$	DAG 34:1	577.521	577.520
**579**	$C_{37}H_{71}O_4$	DAG 34:0	579.540	579.535
**588**	$C_{36}H_{71}NO_3Na$	CerNS18	588.525	588.533
**601**	$C_{39}H_{69}O_4$	DAG 36:3	601.516	601.520
**603**	$C_{39}H_{71}O_4$	DAG 36:2	603.535	603.535
**605**	$C_{39}H_{73}O_4$	DAG 36:1	605.549	605.551
**703**	$C_{39}H_{80}N_2PO_6$	SM 16:0	703.562	703.575
**725**	$C_{39}H_{79}N_2PO_6$ Na	SM 16:0	725.548	725.557
**732**	$C_{40}H_{79}NPO_8$	PC 32:1	732.554	732.554
**734**	$C_{40}H_{81}NPO_8$	PC 32:0	734.562	734.570
**756**	$C_{40}H_{80}NPO_8$ Na / $C_{42}H_{79}NPO_8$	PC32:0 / PC34:3	756.536	756.552 / 756.554
**758**	$C_{42}H_{81}NPO_8$	PC 34:2	758.554	758.570
**760**	$C_{42}H_{83}NPO_8$	PC 34:1	760.577	760.586
**780**	$C_{42}H_{80}NPO_8$ Na	PC 34:2	780.534	780.552
**782**	$C_{42}H_{82}NPO_8$ Na	PC 34:1	782.553	782.568
**784**	$C_{44}H_{83}NPO_8$	PC 36:3	784.565	784.586
**786**	$C_{44}H_{85}NPO_8$	PC 36:2	786.584	786.601
**794**	$C_{42}H_{78}NPO_8$ K	PC 34:3	794.560	794.510
**796**	$C_{42}H_{80}NPO_8$ K	PC 34:2	796.536	796.526

**798**	$C_{42}H_{82}NPO_8$ K	PC 34:1	798.547	798.542
**806**	$C_{44}H_{82}NPO_8$ Na	PC 36:3	806.545	806.568
**808**	$C_{44}H_{84}NPO_8$ Na	PC 36:2	808.566	808.583
**810**	$C_{44}H_{86}NPO_8$ Na	PC 36:1	810.582	810.599

Negative ions

Nominal mass (m/z)	Chemical formula	Molecular assignment	Observed mass (m/z)	Theoretical mass (m/z)
**140**	$C_2H_7NPO_4$	PE fragment	140.026	140.012
**149**	$C_{10}H_{13}O$	Carvacrol	149.100	149.097
**180**	$C_5H_{11}NPO_4$	PE fragment	180.052	180.043
**225**	$C_{14}H_{25}O_2$	C14:1	225.187	225.186
**227**	$C_{14}H_{27}O_2$	C14:0	227.204	227.202
**251**	$C_{16}H_{27}O_2$	C16:2	251.206	251.202
**253**	$C_{16}H_{29}O_2$	C16:1	253.218	253.217
**255**	$C_{16}H_{31}O_2$	C16:0	255.233	255.233
**277**	$C_{18}H_{29}O_2$	C18:3	277.216	277.217
**279**	$C_{18}H_{31}O_2$	C18:2	279.229	279.233
**281**	$C_{18}H_{33}O_2$	C18:1	281.246	281.249
**283**	$C_{18}H_{35}O_2$	C18:0	283.260	283.264
**303**	$C_{20}H_{31}O_2$	C20:4	303.241	303.233
**305**	$C_{20}H_{33}O_2$	C20:3	305.259	305.249
**307**	$C_{20}H_{35}O_2$	C20:2	307.268	307.264
**309**	$C_{20}H_{37}O_2$	C20:1	309.278	309.280
**325**	$C_{21}H_{41}O_2$	C21:0	325.287	325.311
**327**	$C_{22}H_{31}O_2$	C22:6	327.220	327.233
**339**	$C_{22}H_{43}O_2$	C22:0	339.324	339.327
**353**	$C_{23}H_{45}O_2$	C23:0	353.340	353.342
**367**	$C_{24}H_{47}O_2$	C24:0	367.361	367.358
**381**	$C_{25}H_{49}O_2$	C25:0	381.361	381.374
**383**	$C_{27}H_{43}O$	Cholesterol	383.319	383.332
**385**	$C_{27}H_{45}O$	Cholesterol	385.340	385.348
**395**	$C_{26}H_{51}O_2$	C26:0	395.394	395.390
**409**	$C_{27}H_{53}O_2$	C27:0	409.402	409.405
**423**	$C_{28}H_{55}O_2$	C28:0	423.419	423.421
**429**	$C_{29}H_{49}O_2$	Vitamin E	429.343	429.374
**465**	$C_{27}H_{45}SO_4$	Cholesterol sulfate	465.293	465.304
**564**	$C_{36}H_{70}NO_3$	CerNS18	564.500	564.536
**616**	$C_{34}H_{67}NPO_6$	SM 16:0 $(M-C_2H_3N(CH_3)_3)^-$	616.457	616.471
**642**	$C_{36}H_{69}NPO_6$	SM 16:0 $(M-NH(CH_3)_3)^-$	642.475	642.487
**671**	$C_{37}H_{68}PO_8$	PA 34:2	671.467	671.466
**673**	$C_{37}H_{70}PO_8$	PA 34:1	673.469	673.481
**687**	$C_{38}H_{76}N_2PO_6$	SM 16:0 $(M-CH_3)^-$	687.516	687.545
**695**	$C_{39}H_{68}PO_8$	PA 36:4	695.456	695.466
**697**	$C_{39}H_{70}PO_8$	PA 36:3	697.458	697.481
**699**	$C_{39}H_{72}PO_8$	PA 36:2	699.486	699.497
**701**	$C_{39}H_{74}PO_8$	PA 36:1	701.504	701.513

**712**	$C_{39}H_{71}NPO_8$	PE 34:3	712.500	712.492
**714**	$C_{39}H_{73}NPO_8$	PE 34:2	714.508	714.508
**716**	$C_{39}H_{75}NPO_8$	PE 34:1	716.505	716.524
**736**	$C_{41}H_{71}NPO_8$	PE 36:5	736.505	736.492
**738**	$C_{41}H_{73}NPO_8$	PE 36:4	738.512	738.508
**740**	$C_{41}H_{75}NPO_8$	PE 36:3	740.512	740.524
**742**	$C_{41}H_{77}NPO_8$	PE 36:2	742.518	742.539
**744**	$C_{41}H_{79}NPO_8$	PE 36:1	744.527	744.555
**764**	$C_{43}H_{75}NPO_8$	PE 38:5	764.518	764.524
**766**	$C_{43}H_{77}NPO_8$	PE 38:4	766.524	766.539
**768**	$C_{43}H_{79}NPO_8$	PE 38:3	768.538	768.555
**786**	$C_{42}H_{77}NPO_{10}$	PS 36:2	786.523	786.529
**788**	$C_{42}H_{79}NPO_{10}$	PS 36:1	788.537	788.545
**808**	$C_{44}H_{75}NPO_{10}$	PS 38:5	808.499	808.513
**810**	$C_{44}H_{77}NPO_{10}$	PS 38:4	810.512	810.529
**812**	$C_{44}H_{79}NPO_{10}$	PS 38:3	812.538	812.545
**833**	$C_{43}H_{78}PO_{13}$	PI 34:2	833.519	833.519
**835**	$C_{43}H_{80}PO_{13}$	PI 34:1	835.514	835.534
**857**	$C_{45}H_{78}PO_{13}$	PI 36:4	857.513	857.519
**861**	$C_{45}H_{82}PO_{13}$	PI 36:2	861.534	861.550
**885**	$C_{47}H_{82}PO_{13}$	PI 38:4	885.548	885.550

Supplementary Table S2 Results from cutaneous absorption analysis of carvacrol (n=8) and ceramide (n=6) after application of Green Oregano Essential Oil and Ceramide formulations, respectively. Same data as presented also in Figs. 4a and 5a, respectively.

Carvacrol analysis

	Amount (μg/cm²)	% of Applied Dose
**Stratum corneum**	0.071 ± 0.051	0.22 ± 0.15
**Viable epidermis**	0.65 ± 0.21	2.1 ± 0.63
**Dermis**	4.5 ± 2.2	14 ± 6.7
**Receptor fluid**	3.1 ± 2.3	9.8 ± 7.4
**Mass balance**	20 ± 3.4	65 ± 12

Ceramide analysis

	Amount (μg/cm²)	% of Applied Dose
**Stratum corneum**	1.0 ± 0.39	2.1 ± 0.82
**Viable epidermis**	0.47 ± 0.24	0.94 ± 0.42
**Dermis**	0.22 ± 0.33	0.44 ± 0.67
**Receptor fluid**	<0.003	<0.007
**Mass balance**	48 ± 5.0	97 ± 5.4

Supplementary Table S3 Information on skin samples analysed in this study.

Treatment	# of skin samples	# of cross sections	Analysis	Figure nr.
**EO untreated**	3	4	ToF-SIMS (-80°C)+SEM	1, S1
**EO placebo**	2	4	ToF-SIMS (-80°C)+SEM	3, S3
**EO**	3	6	ToF-SIMS (-80°C)+SEM	2, 4, S2, S4
**Ceramide untreated**	1	2	ToF-SIMS (RT)+SEM	5, S5, S6
**Ceramide placebo**	1	2	ToF-SIMS (RT)+SEM	-
**Ceramide**	2	4	ToF-SIMS (RT)+SEM	5, 6, S5, S6, S7, S8
**EO**	8	-	GC-MS	4a
**Ceramide**	6	-	LC-MS	5a

Supplementary Table S4 Composition of formulations used in the studies of carvacrol and ceramide penetration, respectively.

Carvacrol formulation

Raw Materials	Concentration (w/w %)
**EO Green Origano**	1
**Water**	96,3
**ACRYLATES/C10-30 ALKYL ACRYLATE CROSSPOLYMER**	1,4
**AMINOMETHYL PROPANOL**	0,8
**PHENOXYETHANOL**	0,5

Ceramide formulation

Raw Materials	Concentration (w/w %)
**CerNS18**	1
**Water**	72,65
**ISOPROPYL LAUROYL SARCOSINATE**	10
**CYCLOHEXASILOXANE**	5
**ALCOHOL**	5
**GLYCERIN**	3
**ACRYLAMIDE/SODIUM ACRYLOYLDIMETHYLTAURATE COPOLYMER**	1
**ACRYLATES/C10-30 ALKYL ACRYLATE CROSSPOLYMER**	0,5
**CETYL ALCOHOL**	0,4
**PHENOXYETHANOL**	0,4
**GLYCERYL STEARATE + PEG-100 STEARATE**	0,3
**SALICYLIC ACID**	0,3
**METHYLPARABEN**	0,2
**DISODIUM EDTA**	0,15
**ETHYLPARABEN**	0,1

Supplementary Table S5 Carvacrol extraction recovery from different skin layers. Mean ± sd obtained at two different concentration on triplicate

**Compartments**	**Extraction recovery (mean ± sd)**
Washing	100.3 ± 5.4
Strip	94.2 ± 3.7
Viable epidermis	107.7 ± 1.1
Dermis	94.6 ± 4.7
Receptor Fluid	91.2 ± 1.5

Supplementary Table S6 Ceramide extraction recovery from different skin layers. Mean ± sd obtained at two different concentration on triplicate

**Compartments**	**Extraction recovery (mean ± sd)**
Washing	110.97 ± 1.2
Strip	101.21 ± 2.5
Viable epidermis	98.9 ± 7.8
Dermis	92.3 ± 8.0
Receptor Fluid	100.3 ± 4.3

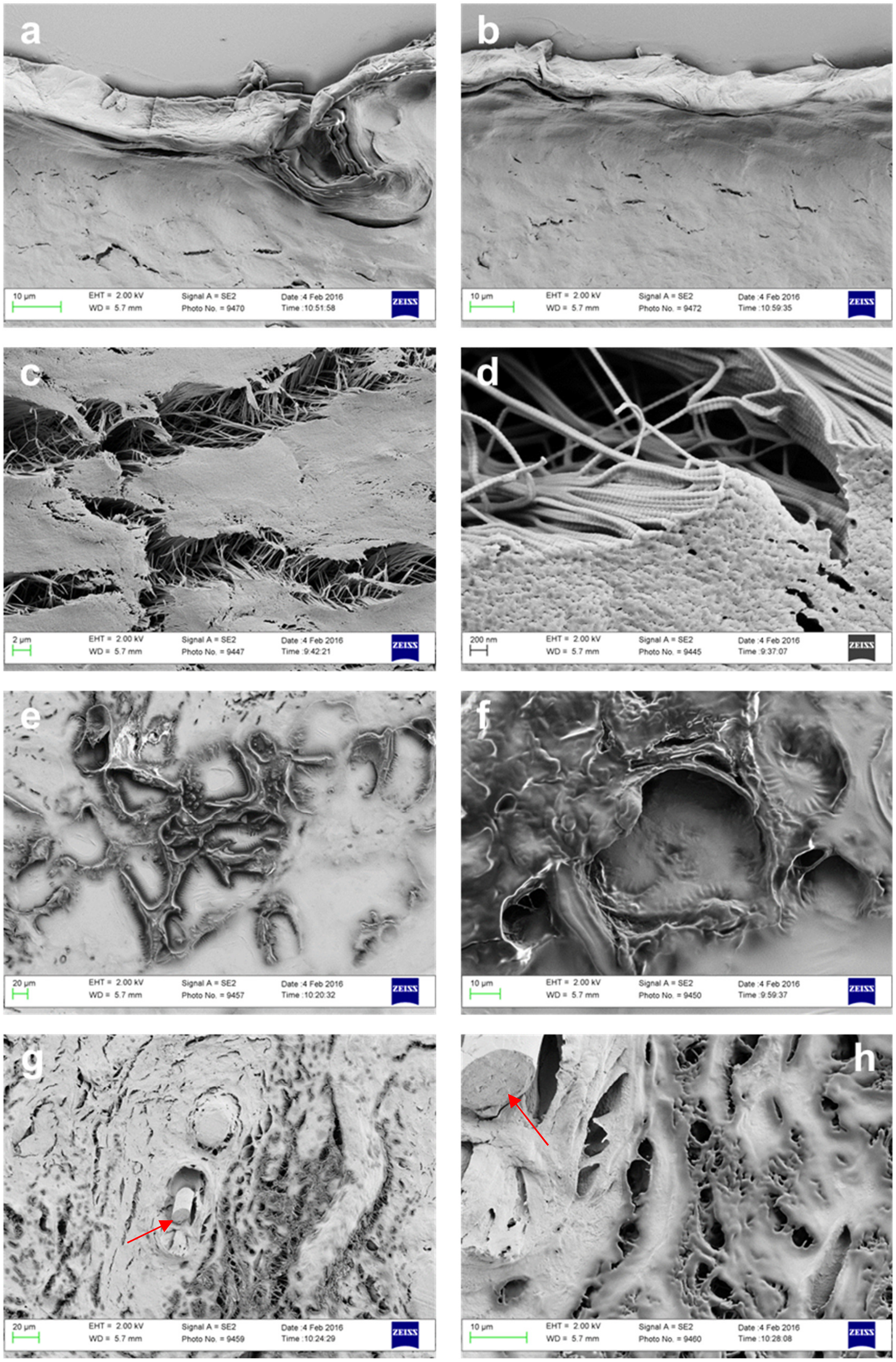

Supplementary Figure S1 SEM images from different regions of the skin cross section displayed in Figure 4, (a-b) epidermis with the corneocyte layers of stratum corneum at the cross section edge, representing the skin surface, (c-d) dermis, note the collagen fibrils with the characteristic 67 nm periodicity, (e-f) fat-rich structures, suggested to correspond to adipocytes, possibly associated with the secretory coil of an eccrine sweat gland, and (g-h) hair follicle (arrows indicate hair fiber).

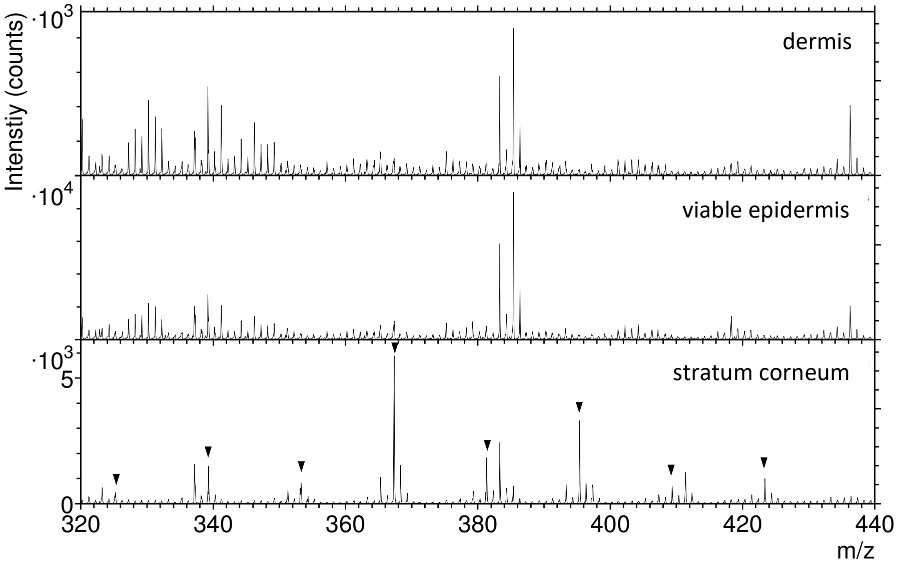

Supplementary Figure S2 Negative ToF-SIMS spectra from regions of interest (ROIs) indicated in Figure 2a. The peaks marked with triangles (stratum corneum) correspond to fatty acid ions C21:0, C22:0, C23:0, C24:0, C25:0, C26:0, C27:0 and C28:0. The peaks at m/z 383 and 385 (dermis and viable epidermis) correspond to cholesterol ions.

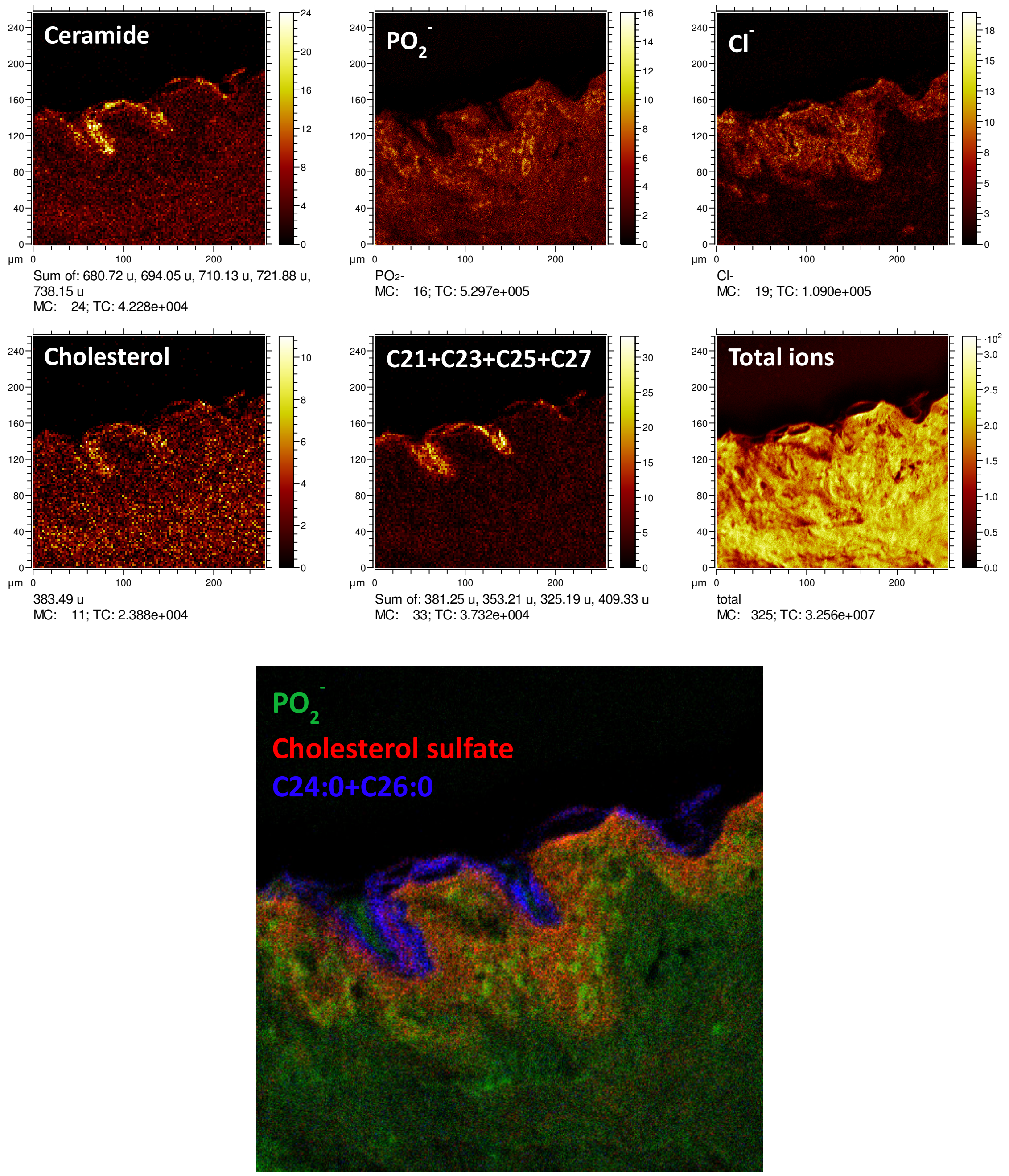

Supplementary Figure S3 High-resolution ToF-SIMS images of the epidermis region of a skin cross section. The peaks used to generate the images are specified below each image, either by their m/z values (u) or the ion identity (PO_2^- and Cl^-, respectively). The bottom image is a 3-colour overlay of the PO_2^- image (green) with the cholesterol sulfate (red) and C24:0+C26:0 (blue) images in Fig. 3. MC – maximum counts per pixel, TC – total counts in image. Field of view 250x250 μm^2. Note that the cholesterol image was generated using only the peak at m/z 383. The molecular ion at m/z 385 showed no localization to SC, possibly indicating a conformation difference between cholesterol in SC and in viable epidermis.

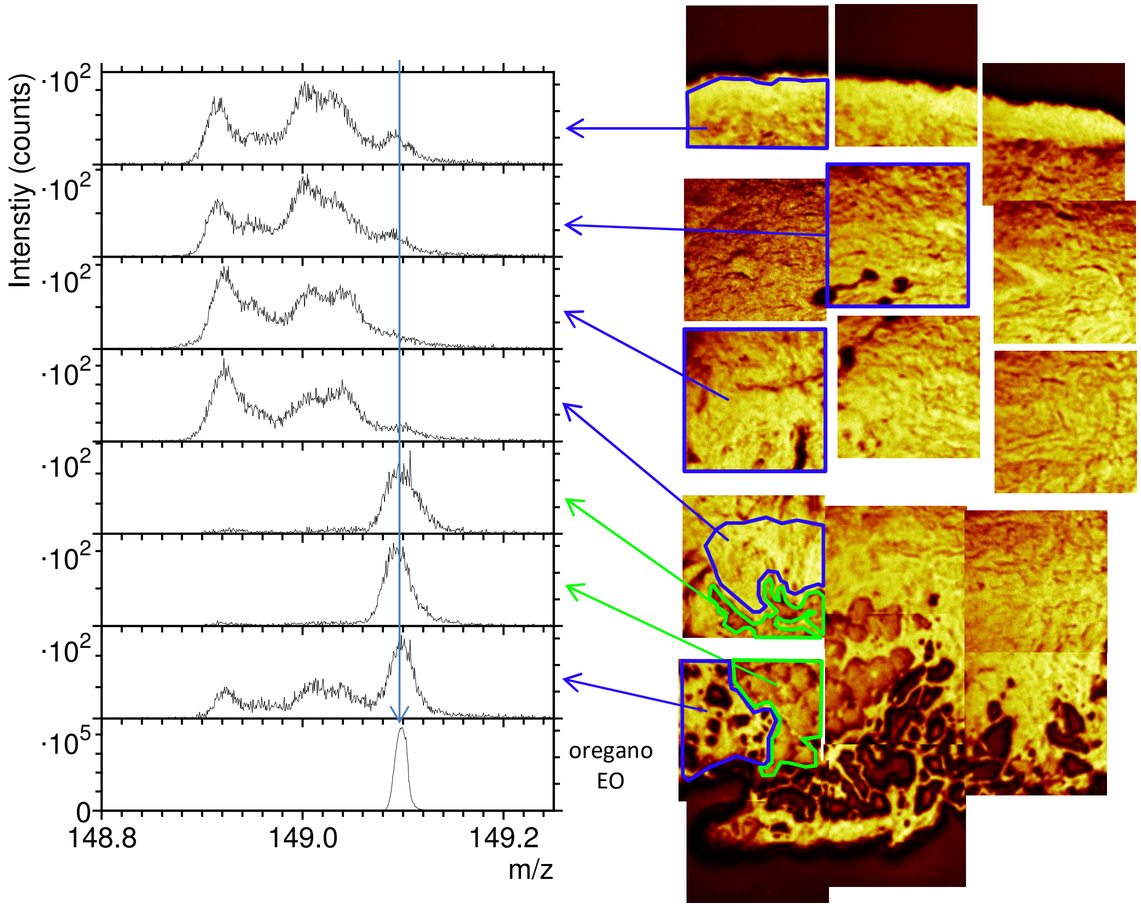

Supplementary Figure S4 ToF-SIMS spectra displaying the carvacrol peak at different locations in skin cross section (same sample as in Figure 4). The spectra were generated from the areas indicated in the total ion images (right), representing regions with low (blue borders) or high (green borders) signal from DAG ions, respectively. The bottom spectrum was measured from pure oregano EO. Note that the spectra have individual intensity scales and that the strength of the carvacrol signal (at m/z 149.10) therefore should be estimated from comparison with the signal of the tissue-related peaks at m/z 148.90 – 149.06.

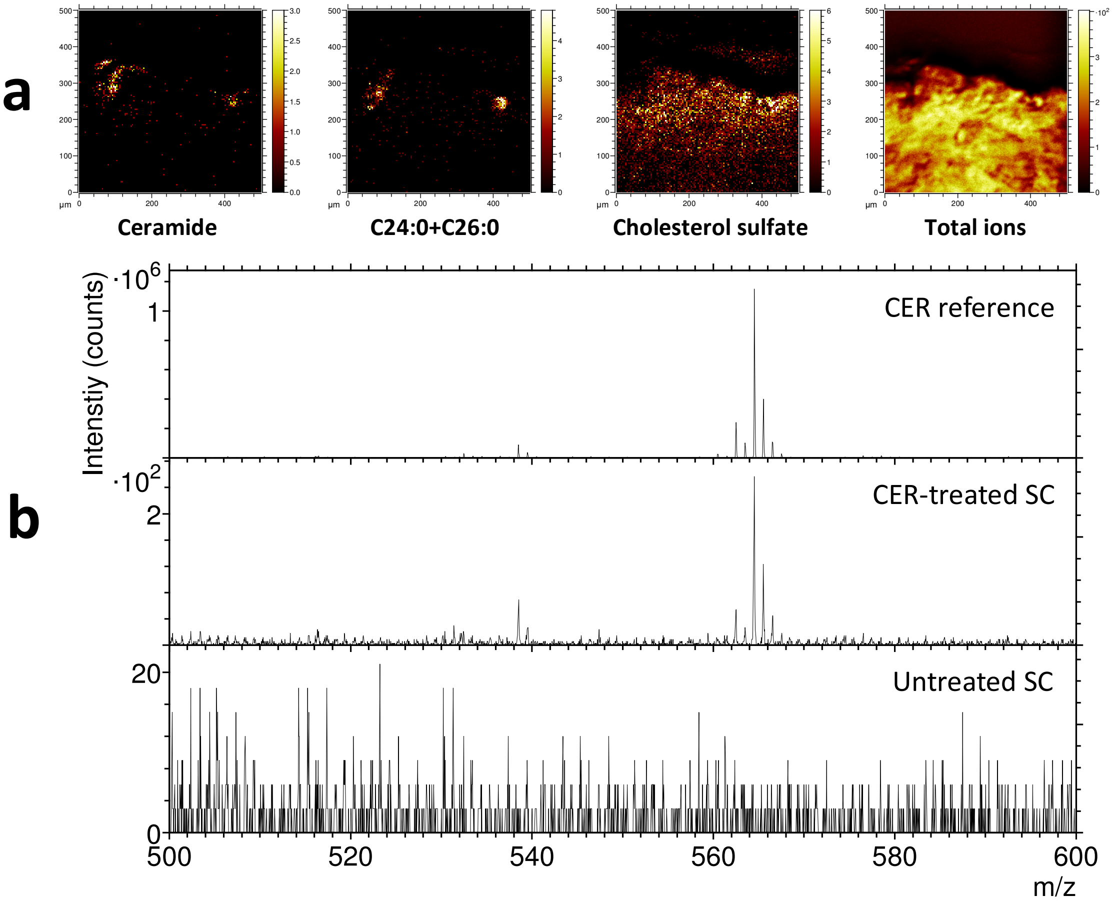

Supplementary Figure S5 Negative ion ToF-SIMS data demonstrating detection of exogeneous ceramide (Cer(d18:1/18:0)) in skin cross sections. (a) Ion images of the epidermis region of a skin sample treated with a ceramide-containing formulation, showing (from left); exogenous ceramide (m/z 564), C24:0+C26:0 (m/z 367+395), cholesterol sulfate (m/z 465), and total ions. Field of view 500x500 μm^2. (b) ToF-SIMS spectra in the mass range of the ceramide molecular ion for a ceramide reference sample (top), the SC region of a skin sample treated with a ceramide-containing formulation (center), and the SC region of an untreated skin sample (bottom). Note the different intensity scales used to demonstrate lack of ceramide signal in the untreated sample.

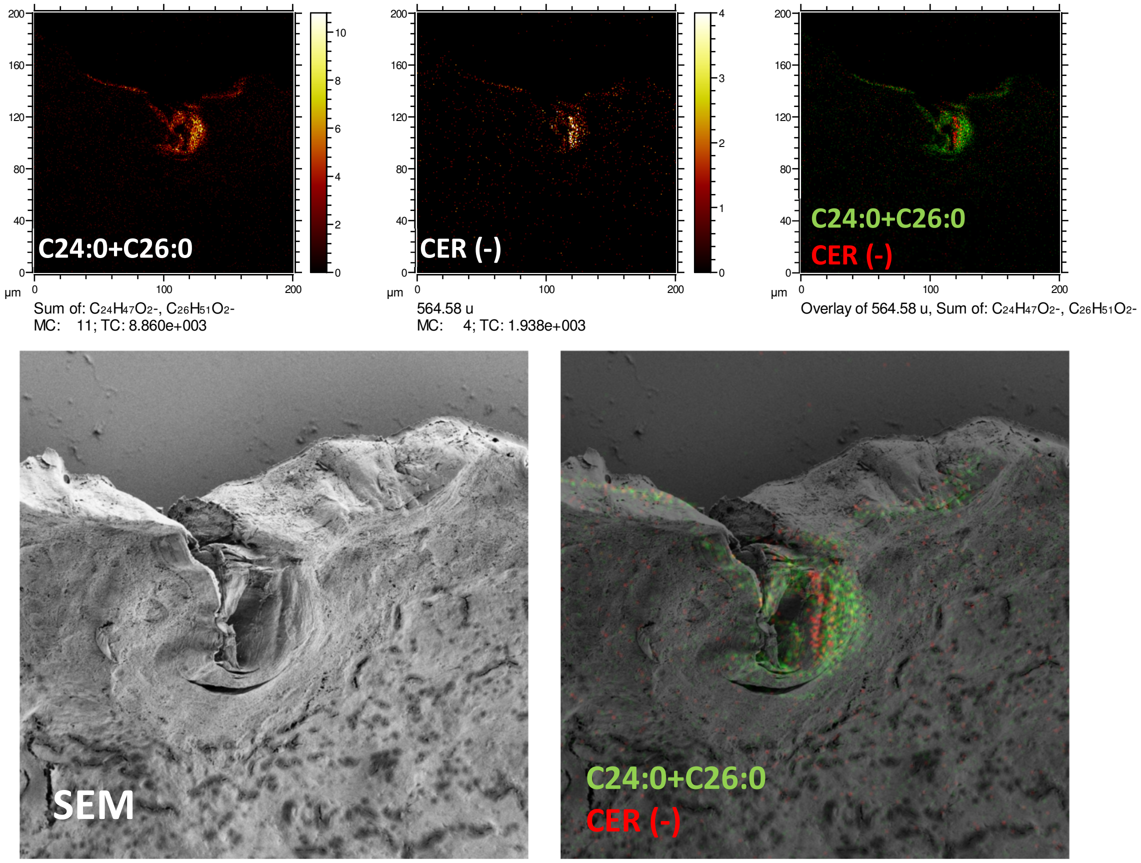

Supplementary Figure S6 High-resolution ToF-SIMS and SEM images of the stratum corneum (SC) region of a skin cross section treated with ceramide formulation. The top row shows negative ion images of C24:0+C26:0 fatty acids (m/z 367+395), exogenous ceramide (m/z 564) and a 2-colour overlay of these two images. The bottom row shows a SEM image of the same analysis area and a superposition of the SEM image and the 2-colour overlay image in the upper row. Note the preferential location of exogenous ceramide to the exposed top surface of stratum corneum, whereas the C24:0+C26:0 fatty acids are mainly located to the deeper layers of SC.

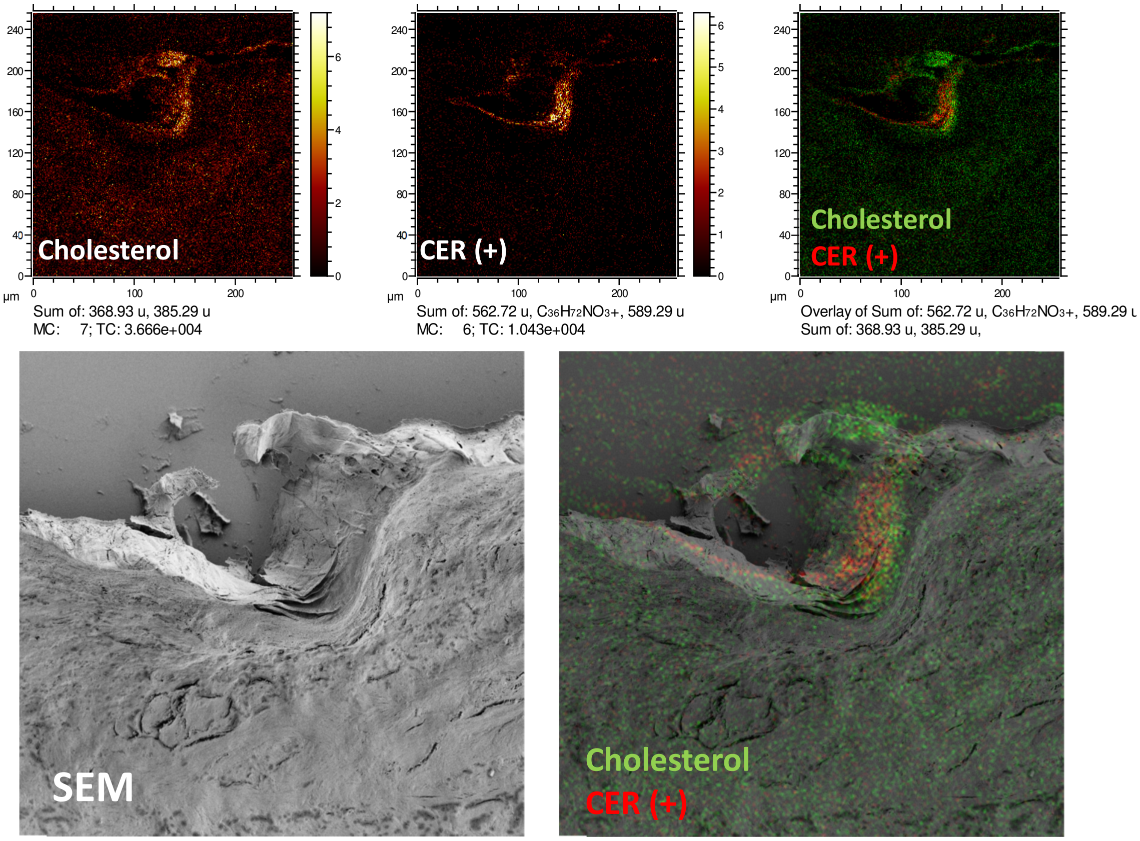

Supplementary Figure S7 High-resolution ToF-SIMS and SEM images of the stratum corneum (SC) region of a skin cross section treated with ceramide formulation. The top row shows positive ion images of cholesterol (m/z 367+395), exogenous ceramide (m/z 562+566+588) and a 2-colour overlay of these two images. The bottom row shows a SEM image of the same analysis area and a superposition of the SEM image and the 2-colour overlay image in the upper row. Note the preferential location of exogenous ceramide to the exposed top surface of stratum corneum, whereas cholesterol shows increased signal from the entire SC area.

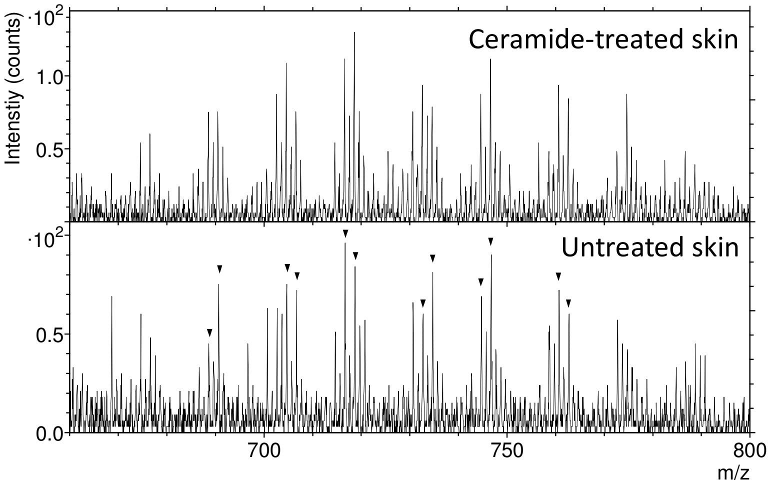

Supplementary Figure S8 Positive ToF-SIMS spectra in the mass range of the endogenous ceramide peaks (indicated by triangles), comparing a ceramide-treated and an untreated skin sample.
